# Childhood generalized specific phobia as an early marker of internalizing psychopathology across the lifespan: results from the World Mental Health Surveys

**DOI:** 10.1186/s12916-019-1328-3

**Published:** 2019-05-24

**Authors:** Ymkje Anna de Vries, Ali Al-Hamzawi, Jordi Alonso, Guilherme Borges, Ronny Bruffaerts, Brendan Bunting, José Miguel Caldas-de-Almeida, Alfredo H. Cia, Giovanni De Girolamo, Rumyana V. Dinolova, Oluyomi Esan, Silvia Florescu, Oye Gureje, Josep Maria Haro, Chiyi Hu, Elie G. Karam, Aimee Karam, Norito Kawakami, Andrzej Kiejna, Viviane Kovess-Masfety, Sing Lee, Zeina Mneimneh, Fernando Navarro-Mateu, Marina Piazza, Kate Scott, Margreet ten Have, Yolanda Torres, Maria Carmen Viana, Ronald C. Kessler, Peter de Jonge, on behalf of the WHO World Mental Health Survey Collaborators, Sergio Aguilar-Gaxiola, Ali Al-Hamzawi, Mohammed Salih Al-Kaisy, Jordi Alonso, Laura Helena Andrade, Corina Benjet, Guilherme Borges, Evelyn J. Bromet, Ronny Bruffaerts, Brendan Bunting, Jose Miguel Caldas de Almeida, Graça Cardoso, Somnath Chatterji, Alfredo H. Cia, Louisa Degenhardt, Koen Demyttenaere, Silvia Florescu, Giovanni de Girolamo, Oye Gureje, Josep Maria Haro, Hristo Hinkov, Chi-yi Hu, Peter de Jonge, Aimee Nasser Karam, Elie G. Karam, Norito Kawakami, Ronald C. Kessler, Andrzej Kiejna, Viviane Kovess-Masfety, Sing Lee, Jean-Pierre Lepine, Daphna Levinson, John McGrath, Maria Elena Medina-Mora, Zeina Mneimneh, Jacek Moskalewicz, Fernando Navarro-Mateu, Beth-Ellen Pennell, Marina Piazza, Jose Posada-Villa, Kate M. Scott, Tim Slade, Juan Carlos Stagnaro, Dan J. Stein, Margreet ten Have, Yolanda Torres, Maria Carmen Viana, Harvey Whiteford, David R. Williams, Bogdan Wojtyniak

**Affiliations:** 1 Faculty of Behavioural and Social Sciences, Department of Developmental Psychology, University of Groningen, Groningen, the Netherlands; 2 Interdisciplinary Center Psychopathology and Emotion regulation, University Medical Center Groningen, University of Groningen, Groningen, the Netherlands; 3 College of Medicine, Al-Qadisiya University, Diwaniya governorate, Iraq; 4 Health Services Research Unit, IMIM-Hospital del Mar Medical Research Institute, Barcelona, Spain; 5 CIBER en Epidemiología y Salud Pública (CIBERESP), Barcelona, Spain; 6 Pompeu Fabra University (UPF), Barcelona, Spain; 7 National Institute of Psychiatry Ramón de la Fuente Muñiz, Mexico City, Mexico; 8 Universitair Psychiatrisch Centrum - Katholieke Universiteit Leuven (UPC-KUL), Campus Gasthuisberg, Leuven, Belgium; 9 School of Psychology, Ulster University, Londonderry, UK; 10 Lisbon Institute of Global Mental Health and Chronic Diseases Research Center (CEDOC), NOVA Medical School / Faculdade de Ciências Médicas, Universidade Nova de Lisboa, Lisbon, Portugal; 11 Anxiety Clinic and Research Center, Buenos Aires, Argentina; 12 IRCCS Istituto Centro San Giovanni di Dio Fatebenefratelli, Brescia, Italy; 13 National Center of Public Health and Analyses, Sofia, Bulgaria; 14 Department of Psychiatry, College of Medicine, University of Ibadan, Ibadan, Nigeria; 15 National School of Public Health, Management and Development, Bucharest, Romania; 16 Department of Psychiatry, University College Hospital, Ibadan, Nigeria; 17 Parc Sanitari Sant Joan de Déu, CIBERSAM, Universitat de Barcelona, Sant Boi de Llobregat, Barcelona, Spain; 18 Department of Psychology, College of Education, King Saud University, Riyadh, Saudi Arabia; 19 Shenzhen Institute of Mental Health and Shenzhen Kangning Hospital, Shenzhen, China; 20 Department of Psychiatry and Clinical Psychology, Faculty of Medicine, Balamand University, Beirut, Lebanon; 21 Department of Psychiatry and Clinical Psychology, St George Hospital University Medical Center, Beirut, Lebanon; 22 Institute for Development Research Advocacy and Applied Care (IDRAAC), Beirut, Lebanon; 23 National Institute of Mental Health, National Center for Neurology and Psychiatry, Kodaira, Tokyo Japan; 24 Wroclaw Medical University, Wrocław, Poland; 25 University of Lower Silesia, Wroclaw, Poland; 26 Ecole des Hautes Etudes en Santé Publique (EHESP), EA 4057, Paris Descartes University, Paris, France; 27 Department of Psychiatry, Chinese University of Hong Kong, Tai Po, Hong Kong; 28 Survey Research Center, Institute for Social Research, University of Michigan, Ann Arbor, MI USA; 29 UDIF-SM, Subdirección General de Planificación, Innovación y Cronicidad, Servicio Murciano de Salud, IMIB-Arrixaca, CIBERESP-Murcia, Murcia, Spain; 30 Instituto Murciano de Investigación Biosanitaria (IMIB) Virgen de la Arrixaca, Murcia, Spain; 31 Centro de Investigación Biomédica en ERed en Epidemiología y Salud Pública (CIBERESP), Murcia, Spain; 32 Instituto Nacional de Salud, Lima, Peru; 33 Universidad Cayetano Heredia, Lima, Peru; 34 Department of Psychological Medicine, University of Otago, Dunedin, Otago New Zealand; 35 Trimbos Instituut, Netherlands Institute of Mental Health and Addiction, Utrecht, Netherlands; 36 Center for Excellence on Research in Mental Health, CES University, Medellin, Colombia; 37 Department of Social Medicine, Postgraduate Program in Public Health, Federal University of Espírito Santo, Vitoria, Brazil; 38 Department of Health Care Policy, Harvard Medical School, Boston, MA USA

**Keywords:** Specific phobia, Internalizing disorders, Early markers, Comorbidity, Suicidality

## Abstract

**Background:**

Specific phobia (SP) is a relatively common disorder associated with high levels of psychiatric comorbidity. Because of its early onset, SP may be a useful early marker of internalizing psychopathology, especially if generalized to multiple situations. This study aimed to evaluate the association of childhood generalized SP with comorbid internalizing disorders.

**Methods:**

We conducted retrospective analyses of the cross-sectional population-based World Mental Health Surveys using the Composite International Diagnostic Interview. Outcomes were lifetime prevalence, age of onset, and persistence of internalizing disorders; past-month disability; lifetime suicidality; and 12-month serious mental illness. Logistic and linear regressions were used to assess the association of these outcomes with the number of subtypes of childhood-onset (< 13 years) SP.

**Results:**

Among 123,628 respondents from 25 countries, retrospectively reported prevalence of childhood SP was 5.9%, 56% of whom reported one, 25% two, 10% three, and 8% four or more subtypes. Lifetime prevalence of internalizing disorders increased from 18.2% among those without childhood SP to 46.3% among those with one and 75.6% those with 4+ subtypes (OR = 2.4, 95% CI 2.3–2.5, *p* < 0.001). Twelve-month persistence of lifetime internalizing comorbidity at interview increased from 47.9% among those without childhood SP to 59.0% and 79.1% among those with 1 and 4+ subtypes (OR = 1.4, 95% CI 1.4–1.5, *p* < 0.001). Respondents with 4+ subtypes also reported significantly more disability (3.5 days out of role in the past month) than those without childhood SP (1.1 days) or with only 1 subtype (1.8 days) (*B* = 0.56, SE 0.06, *p* < 0.001) and a much higher rate of lifetime suicide attempts (16.8%) than those without childhood SP (2.0%) or with only 1 subtype (6.5%) (OR = 1.7, 95% CI 1.7–1.8, *p* < 0.001).

**Conclusions:**

This large international study shows that childhood-onset generalized SP is related to adverse outcomes in the internalizing domain throughout the life course. Comorbidity, persistence, and severity of internalizing disorders all increased with the number of childhood SP subtypes. Although our study cannot establish whether SP is causally associated with these poor outcomes or whether other factors, such as a shared underlying vulnerability, explain the association, our findings clearly show that childhood generalized SP identifies an important target group for early intervention.

**Electronic supplementary material:**

The online version of this article (10.1186/s12916-019-1328-3) contains supplementary material, which is available to authorized users.

## Introduction

Anxiety and mood disorders are major contributors to the global disease burden [1] due partly to their high prevalence [2], early onset [3], and chronic or recurrent course [4–6]. Although much effort has been devoted to improving the course of these disorders, treatment is still insufficient to avert most of the burden [7–11]. Consequently, prevention may be a necessary alternative strategy.

Comorbidity between mental disorders is common [12, 13], which has led theorists to posit a latent structure of psychopathology, reducing a variety of disorders to a limited set of domains [14]. These domains, of which the internalizing and externalizing have been confirmed most frequently [15–20], are thought to represent core psychopathological processes underlying the varied clinical manifestations of disorders. If so, targeting these underlying processes might offer new opportunities for prevention. Internalizing disorders, for example, develop at very different ages: specific phobia (SP) has a median age of onset at 8 years old [21], whereas major depressive disorder (MDD) and generalized anxiety disorder (GAD) have median onset at 30–40 years old [3, 22]. If interventions could successfully treat the earlier disorders in this domain, this might lead to reductions in the subsequent onset, persistence, or severity of other disorders in the same domain.

Although SP is often considered a relatively mild disorder [23, 24], the structure of psychopathology model suggests that it might identify persons with a vulnerability for more serious later disorders in the same domain. In support of this possibility, SP has been associated with increased risk of later-onset disorders in the internalizing domain [21, 25–34]. Importantly, persons with SP may have only a single fear, or they may have many (e.g., of spiders, storms, blood, and heights). Some evidence suggests that persons with multiple fears have a greater risk of comorbidity and impairment [21, 29, 33, 35, 36], suggesting that g*eneralized* SP may be a marker of particularly high internalizing vulnerability.

With an eye toward early intervention, SP *with an onset in childhood* is of greatest interest, but to date, no study has examined internalizing comorbidity in this group of people. Furthermore, information about other important aspects of comorbidity, such as age of onset, persistence, and severity, and other outcomes, such as suicidality, is largely lacking. In this paper, we therefore examine these outcomes. We focus on the number of phobia subtypes as a marker of generalization of the underlying psychopathological process, expecting worse outcomes among persons reporting more fears.

## Methods

### Survey samples

Data came from the World Mental Health Surveys (WMHS), which include cross-sectional surveys administered in low/lower-middle-income countries, upper-middle-income countries, and high-income countries (Additional file 1: Table S1). Adults were selected using probability sampling methods designed to generate population representative samples, and interviews were conducted face to face in respondent homes. A total of 123,628 respondents from 25 countries participated in the present study. Informed consent was obtained according to protocols endorsed by local Institutional Review Boards. Within-country sampling methods are described in detail elsewhere [37, 38].

### Measures

#### Mental disorders

Lifetime and 12-month mental disorders were assessed with the WHO Composite International Diagnostic Interview (CIDI) [39]. To reduce respondent burden, interviews were administered in two parts. All respondents completed part I, assessing core mental disorders. Part II, assessing other disorders and correlates, was administered to all respondents with any lifetime part I diagnosis and a probability subsample of other part I respondents. Part II data were weighted so that weighted prevalence estimates are identical to those in the part I sample.

We included the following internalizing disorders: anxiety disorders (panic disorder, agoraphobia, GAD, social anxiety disorder, SP, post-traumatic stress disorder [PTSD], separation anxiety disorder), mood disorders (MDD and/or dysthymia, bipolar disorder [I, II, or subthreshold]), and eating disorders (bulimia nervosa, binge eating disorder). CIDI diagnoses have shown generally good concordance with diagnoses based on blinded Structured Clinical Interview for DSM-IV (SCID) reappraisal [40]. Age of onset was assessed with retrospective reports. Persistence was defined as the presence of the disorder in the 12 months before interview among lifetime cases.

#### Specific phobia

The CIDI distinguishes between six SP subtypes: animals, still water or weather, high places, blood-injection-injury, closed spaces, and flying. For each participant, we determined how many subtypes with an age of onset prior to age 13 were reported. Because few participants reported more than four subtypes, we collapsed participants reporting four or more subtypes into a single group. Participants who developed SP later in life were included in the group of participants without childhood SP.

#### Impairment and suicidality

Severe role impairment due to SP was assessed with a modified Sheehan Disability Scale (SDS) [41]. Respondents with 12-month SP rated its interference with functioning in four domains (home management, work, close relationships, and social life) on a scale of 0–10 during the worst month in the past year. Severe impairment was defined as a score ≥ 7 in at least one domain. Respondents with lifetime SP were asked whether they had ever sought treatment specifically for SP.

All respondents were asked how many days in the past 30 days they were totally unable to work or carry out normal activities because of any physical or mental health problems, whether they had ever seriously thought about committing suicide, and, if so, whether they had ever made a plan or attempted suicide. We also examined serious mental illness (SMI), which was defined as meeting criteria for 12-month bipolar I disorder or having another 12-month mood or anxiety disorder (other than SP) with either severe role impairment or a past-year suicide attempt [42].

#### Statistical analysis

We tested the linear association of the number of early-onset SP subtypes, both within the whole sample and within the subsample with childhood SP, using logistic regression for dichotomous variables and linear regression for continuous variables (SAS 9.4). We used the actuarial method to determine the age of onset of comorbid disorders and tested for differences in age of onset depending on the number of SP subtypes using discrete-time logistic regression in the subsample with the comorbid disorder. We also used the actuarial method to calculate the projected lifetime risk of any internalizing disorder, which takes into account that respondents who have not had a disorder yet may still develop the disorder later in life. We also estimated population attributable fractions (PAFs) [43], which indicate the fraction of an outcome in the population that is attributable to childhood SP (assuming a causal relationship, noting that the latter can only be confirmed experimentally). All analyses controlled for country of origin of the participant. Because the data were clustered and weighted, standard errors were estimated using the Taylor series linearization method (SUDAAN 11.0.1). Significance tests were evaluated using *α* = 0.005 (two-sided) to reduce the chance of false-positive findings given the many tests performed.

As sensitivity analyses, we tested interactions between the number of SP subtypes and age group (18–34, 35–49, and 50+ years old), to examine whether associations between childhood phobia and mental health outcomes persisted into older adulthood. We also tested whether associations were found for participants with and without current internalizing psychopathology and for participants from high-income countries as well as those from low- or middle-income countries.

### Results

### Specific phobia

Out of 123,628 participants, 51.8% were female and the mean age was 42.0 (SD = 16.9). Lifetime and 12-month SP prevalence was 7.6% and 5.7%, respectively. Most respondents reported onset before the age of 13, resulting in a 5.9% lifetime prevalence of childhood-onset SP. Prevalence was 3.3% for one early-onset subtype, 1.5% for two, 0.6% for three, and 0.5% for 4+ (Table 1). Persistence was high and increased with number of subtypes (from 73.4% for participants with one subtype to 85.2% for those with 4+ subtypes, OR = 1.2, *p* < 0.001). Severe impairment due to SP and treatment for SP were uncommon (17.5–26.5%), but increased with an increasing number of subtypes (OR = 1.1–1.3, *p* = 0.004 and < 0.001).

**Table 1:** Prevalence and characteristics of specific phobia

	Any specific phobia	Any early-onset specific phobia	Number of subtypes				Test of linear effect	
			1	2	3	4+		
	% (SE)	% (SE)	% (SE)	% (SE)	% (SE)	% (SE)	OR (95% CI)	*p* value
Lifetime prevalence	7.6 (0.1)	5.9 (0.1)	3.3 (0.1)	1.5 (0.0)	0.6 (0.0)	0.5 (0.0)	–	–
12-month prevalence	5.7 (0.1)	4.4 (0.1)	2.4 (0.1)	1.1 (0.0)	0.5 (0.0)	0.4 (0.0)	–	–
Persistence	74.3 (0.6)	75.2 (0.7)	73.4 (0.9)	75.2 (1.3)	77.6 (1.8)	85.2 (1.8)	1.2* (1.1–1.3)	< 0.001
Severe disability	19.1 (0.6)	19.7 (0.6)	17.5 (0.8)	20.2 (1.4)	24.0 (2.0)	26.5 (2.4)	1.3* (1.1–1.4)	< 0.001
Treatment for specific phobia	22.4 (0.5)	20.8 (0.6)	19.1 (0.8)	22.9 (1.2)	22.4 (1.7)	22.8 (2.0)	1.1* (1.0–1.2)	0.004

*p < 0.005

### Internalizing disorder comorbidity

**Fig. 1:**
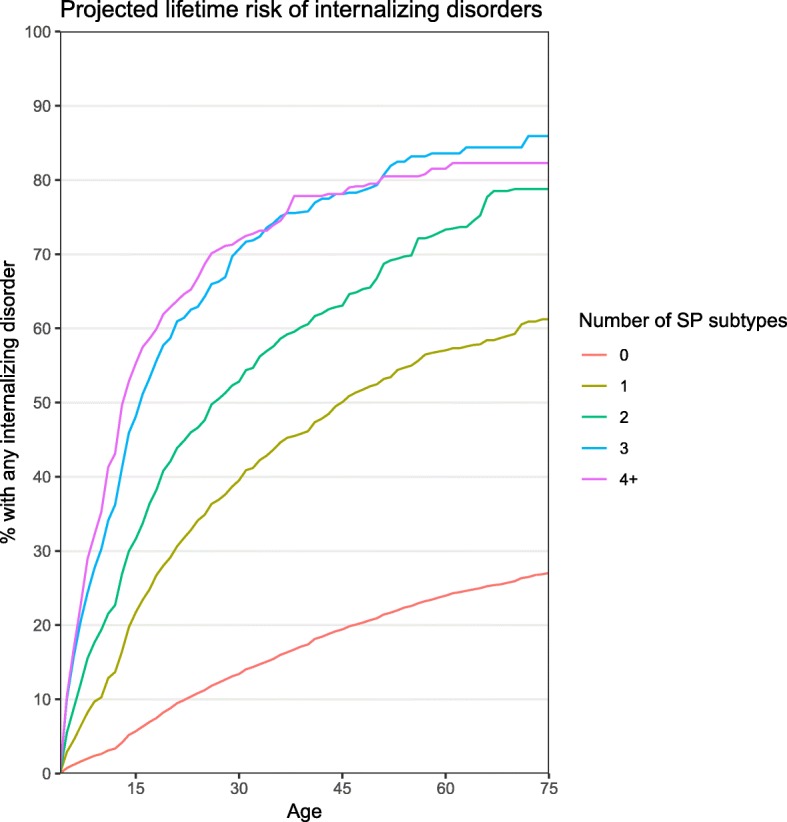
Projected risk of any internalizing disorder by age 75, by number of childhood specific phobia subtypes. Projected risk was calculated using the actuarial method and accounts for censoring of participants who have not yet reached the age of 75 by the time of the interview

Lifetime prevalence of any internalizing disorder increased from 18.2% among those without childhood SP to 46.3% among those with one SP subtype and 75.6% among those with 4+ subtypes (OR = 2.4 in the total sample and 1.6 in the SP subsample, *p* < 0.001) (Table 2). Figure 1 shows the projected risk of any internalizing disorder by age and number of SP subtypes. Examining separate disorder groupings, similar patterns were apparent for anxiety disorders (OR = 2.4 (total sample) and 1.6 (SP subsample), *p* < 0.001), mood disorders (OR = 1.9 (total sample) and 1.4 (SP subsample), *p* < 0.001), and eating disorders (OR = 1.8 (total sample) and 1.3 (SP subsample), *p* < 0.001 and *p* = 0.003). Only 0.6% of respondents without SP and 4.2% of those with one SP subtype met criteria for 4+ internalizing disorders (other than SP), compared to 19.4% of respondents with 4+ SP subtypes (OR = 2.6 (total sample) and 1.7 (SP subsample), *p* < 0.001). SP preceded other internalizing disorders in 79.4% of comorbid cases; in an additional 10.8% of cases, the onset of SP and another internalizing disorder coincided.

**Table 2:** Prevalence of comorbid internalizing disorders, as a function of the number of specific phobia subtypes

Comorbid disorder	Number of subtypes					Test of linear effect (total sample)		Test of linear effect (SP cases only)	
	0	1	2	3	4+				
	% (SE)	% (SE)	% (SE)	% (SE)	% (SE)	OR (95% CI)	*p* value	OR (95% CI)	*p* value
Agoraphobia	0.8 (0.0)	5.1 (0.4)	9.5 (0.8)	17.4 (2.0)	21.2 (2.3)	2.6* (2.4–2.7)	< 0.001	1.7* (1.5–1.9)	< 0.001
Generalized anxiety disorder	3.5 (0.1)	9.7 (0.6)	14.2 (1.1)	21.0 (1.8)	21.3 (2.6)	1.8* (1.7–1.9)	< 0.001	1.4* (1.3–1.5)	< 0.001
Panic disorder	1.4 (0.0)	5.5 (0.5)	8.8 (0.7)	12.6 (1.5)	15.8 (1.8)	2.0* (1.9–2.1)	< 0.001	1.5* (1.3–1.6)	< 0.001
Post-traumatic stress disorder	2.9 (0.1)	9.0 (0.6)	15.3 (1.2)	18.5 (1.8)	22.2 (2.3)	1.9* (1.8–2.0)	< 0.001	1.4* (1.3–1.6)	< 0.001
Separation anxiety disorder	3.9 (0.1)	12.7 (0.9)	19.7 (1.7)	27.2 (2.5)	27.4 (2.7)	1.9* (1.8–2.0)	< 0.001	1.4* (1.2–1.5)	< 0.001
Social anxiety disorder	3.3 (0.1)	14.9 (0.8)	23.2 (1.3)	31.3 (2.3)	38.2 (2.8)	2.3* (2.2–2.4)	< 0.001	1.5* (1.4–1.6)	< 0.001
*Any anxiety disorder*	*10.5 (0.2)*	*33.6 (1.1)*	*47.0 (1.7)*	*62.4 (2.3)*	*66.1 (2.8)*	2.4* (2.3–2.5)	*< 0.001*	1.6* (1.5–1.7)	*< 0.001*
Major depression/dysthymia	10.5 (0.2)	24.1 (0.9)	30.1 (1.4)	36.2 (2.5)	34.1 (2.7)	1.6* (1.6–1.7)	< 0.001	1.2* (1.2–1.3)	< 0.001
Bipolar disorder	1.8 (0.1)	5.7 (0.5)	10.0 (0.9)	12.7 (1.5)	17.3 (1.9)	1.9* (1.8–2.0)	< 0.001	1.5* (1.3–1.6)	< 0.001
*Any mood disorder*	*12.0 (0.2)*	*28.8 (1.0)*	*39.3 (1.5)*	*47.8 (2.5)*	*50.9 (3.0)*	1.9* (1.8–1.9)	*< 0.001*	1.4* (1.3–1.5)	*< 0.001*
Bulimia nervosa	0.6 (0.0)	2.0 (0.3)	3.6 (0.7)	6.8 (1.6)	5.9 (1.6)	2.0* (1.8–2.2)	< 0.001	1.7* (1.4–2.0)	< 0.001
Binge eating disorder	1.4 (0.1)	5.7 (0.8)	5.3 (0.8)	6.6 (1.7)	6.3 (1.7)	1.7* (1.5–1.8)	< 0.001	1.0 (0.8–1.3)	0.746
*Any eating disorder*	*1.9 (0.1)*	*7.2 (0.9)*	*8.3 (1.0)*	*12.4 (2.0)*	*11.4 (2.2)*	1.8* (1.7–1.9)	*< 0.001*	1.3* (1.1–1.5)	*0.003*
*Any internalizing disorder*	*18.2 (0.2)*	*46.3 (1.2)*	*61.1 (1.7)*	*75.6 (2.1)*	*75.6 (2.7)*	2.4* (2.3–2.5)	*< 0.001*	1.6* (1.5–1.8)	*< 0.001*
Exactly 1 internalizing disorder	12.3 (0.2)	24.0 (1.0)	27.6 (1.5)	26.8 (2.2)	21.6 (2.4)	1.4* (1.3–1.4)	< 0.001	1.0 (0.9–1.1)	0.677
Exactly 2 internalizing disorders	4.0 (0.1)	12.2 (0.7)	15.2 (1.2)	21.6 (2.0)	19.7 (2.2)	1.7* (1.6–1.8)	< 0.001	1.2* (1.1–1.3)	< 0.001
Exactly 3 internalizing disorders	1.4 (0.0)	5.9 (0.5)	9.2 (0.8)	12.8 (1.5)	14.9 (1.8)	2.0* (1.9–2.1)	< 0.001	1.4* (1.2–1.5)	< 0.001
4+ internalizing disorders	0.6 (0.0)	4.2 (0.4)	9.0 (0.8)	14.4 (1.7)	19.4 (2.6)	2.6* (2.4–2.8)	< 0.001	1.7* (1.6–2.0)	< 0.001

Italics indicate the main groups of disorders. *p < 0.005

Estimated PAFs for any childhood SP ranged from 8.7% for MDD to 38.8% for agoraphobia (Additional file 1: Table S2). This means that if the associations found here are accurate reflections of causal effects either of childhood SP or of a latent liability that could be successfully treated by early intervention with childhood SP, the population-level lifetime prevalence of these disorders would be expected to decrease proportionally by 8.7–38.8%. The PAFs for any internalizing disorder and for 4+ internalizing disorders were 10.2% and 39.4%, respectively.

Persistence of any lifetime internalizing disorder increased from 47.9% for respondents without childhood SP to 59.0% for those with one SP subtype and 79.1% for those with 4+ subtypes (OR = 1.4 (total sample) and 1.3 (SP subsample), *p* < 0.001) (Table 3). Examining separate disorder groupings, similar patterns were found for anxiety disorders (OR = 1.4 (total sample) and 1.2 (SP subsample), *p* < 0.001) and mood disorders (OR = 1.3 (total sample) and 1.2 (SP subsample), *p* < 0.001), but not for eating disorders (OR = 1.1 (total sample) and 0.8 (SP subsample), *p* = 0.164–0.295).

**Table 3:** Persistence of comorbid internalizing disorders, as a function of the number of specific phobia subtypes

Comorbid disorder	Number of subtypes					Test of linear effect (total sample)		Test of linear effect (SP cases only)	
	0	1	2	3	4+				
	% (SE)	% (SE)	% (SE)	% (SE)	% (SE)	OR (95% CI)	*p* value	OR (95% CI)	*p* value
Agoraphobia	57.8 (2.6)	67.6 (3.9)	64.6 (5.1)	63.6 (6.6)	86.4 (3.2)	1.3* (1.1–1.4)	< 0.001	1.3* (1.1–1.6)	0.002
Generalized anxiety disorder	46.2 (1.1)	52.3 (3.0)	56.3 (3.7)	51.0 (4.7)	68.7 (5.3)	1.2* (1.1–1.3)	< 0.001	1.1 (1.0–1.3)	0.155
Panic disorder	56.1 (1.9)	59.3 (4.1)	65.7 (3.9)	63.6 (5.8)	80.7 (4.7)	1.2* (1.1–1.4)	< 0.001	1.3* (1.1–1.6)	0.003
Post-traumatic stress disorder	46.0 (1.5)	53.4 (3.5)	56.0 (4.0)	59.6 (4.9)	50.9 (5.4)	1.2* (1.0–1.3)	0.003	1.0 (0.9–1.2)	0.821
Separation anxiety disorder	20.4 (1.3)	20.3 (3.1)	24.2 (3.1)	23.3 (4.2)	26.3 (4.3)	1.1 (1.0–1.2)	0.109	1.1 (0.9–1.3)	0.345
Social anxiety disorder	56.2 (1.3)	65.3 (2.7)	73.9 (2.8)	73.4 (3.6)	80.2 (3.5)	1.3* (1.2–1.5)	< 0.001	1.2 (1.0–1.3)	0.027
*Any anxiety disorder*	*48.9 (0.8)*	*59.5 (1.8)*	*65.4 (2.1)*	*65.5 (2.9)*	*76.0 (2.9)*	1.4* (1.3–1.4)	*< 0.001*	1.2* (1.1–1.3)	*< 0.001*
Major depression/dysthymia	39.7 (0.6)	48.8 (2.0)	51.5 (2.7)	56.3 (3.8)	63.3 (4.5)	1.3* (1.2–1.4)	< 0.001	1.2 (1.0–1.3)	0.007
Bipolar disorder	56.7 (1.6)	57.4 (4.3)	67.9 (4.8)	70.2 (5.4)	77.5 (4.9)	1.2* (1.1–1.4)	< 0.001	1.4* (1.2–1.8)	0.001
*Any mood disorder*	*41.9 (0.5)*	*50.2 (1.8)*	*55.3 (2.4)*	*59.7 (3.1)*	*68.0 (3.3)*	1.3* (1.2–1.4)	*< 0.001*	1.2* (1.1–1.4)	*< 0.001*
*Any eating disorder*	*42.9 (2.3)*	*54.0 (5.7)*	*55.7 (5.7)*	*38.1 (8.8)*	*45.1 (8.9)*	*1.1 (0.9–1.2)*	*0.295*	*0.8 (0.6–1.1)*	*0.164*
*Any internalizing disorder*	*47.9 (0.5)*	*59.0 (1.5)*	*66.1 (2.0)*	*70.5 (2.5)*	*79.1 (2.5)*	1.4* (1.4–1.5)	*< 0.001*	1.3* (1.2–1.4)	*< 0.001*

Persistence is defined as the presence of a 12-month disorder among lifetime cases with that disorder. Because of low prevalence, persistence of individual eating disorders was not assessed

Italics indicate the main groups of disorders. *p < 0.005

Median age of onset of comorbid internalizing disorders generally decreased with increasing number of early-onset SP subtypes (Table 4), especially for agoraphobia, GAD, panic disorder, and MDD/dysthymia. For GAD, median age of onset was 39 for those without childhood SP, 37 for those with one subtype, and 28 for those with 4+ subtypes (OR = 1.2 (total sample and SP subsample), *p* < 0.001). Similarly, median age of onset decreased from 21 to 12 for agoraphobia, from 33 to 18 for panic disorder, and from 38 to 29 for MDD/dysthymia (OR = 1.2 (total sample) and 1.1–1.3 (SP subsample), *p* < 0.001). For social anxiety disorder, there was also a consistent, though slight, decrease in age of onset with an increasing number of SP subtypes (from 14 to 12, OR = 1.2 (total sample) and 1.3 (SP subsample), *p* < 0.001). For PTSD, separation anxiety disorder, and bipolar disorder, patterns were less consistent (OR = 1.0–1.2, *p* < 0.001–0.235).

**Table 4:** Age of onset of comorbid disorders, as a function of the number of specific phobia subtypes

Comorbid disorder	Number of subtypes					Test of linear effect (whole sample)		Test of linear effect (SP cases only)	
	0	1	2	3	4+				
	Median (IQR)	Median (IQR)	Median (IQR)	Median (IQR)	Median (IQR)	OR (95% CI)	*p* value	OR (95% CI)	*p* value
Agoraphobia	21 (13–35)	21 (13–39)	20 (13–40)	16 (8–31)	12 (8–17)	1.2* (1.1–1.2)	< 0.001	1.3* (1.2–1.4)	< 0.001
Generalized anxiety disorder	39 (26–54)	37 (24–52)	35 (21–52)	33 (20–44)	28 (18–46)	1.2* (1.1–1.2)	< 0.001	1.2* (1.1–1.2)	< 0.001
Panic disorder	33 (21–49)	29 (19–41)	35 (18–60)	24 (14–41)	18 (11–27)	1.2* (1.2–1.3)	< 0.001	1.2* (1.1–1.3)	< 0.001
Post-traumatic stress disorder	36 (21–54)	33 (20–49)	36 (19–53)	35 (19–75)	33 (19–64)	1.1* (1.0–1.1)	0.002	1.1 (1.0–1.2)	0.166
Separation anxiety disorder	19 (10–31)	19 (8–29)	18 (8–30)	14 (8–36)	17 (8–28)	1.0 (1.0–1.1)	0.074	1.0 (1.0–1.1)	0.235
Social anxiety disorder	14 (9–18)	13 (10–16)	13 (8–16)	13 (8–17)	12 (7–15)	1.2* (1.1–1.2)	< 0.001	1.3* (1.3–1.4)	< 0.001
Major depression/dysthymia	38 (25–53)	33 (22–48)	34 (21–51)	30 (18–50)	29 (15–46)	1.2* (1.1–1.2)	< 0.001	1.1* (1.0–1.2)	0.001
Bipolar disorder	25 (18–38)	26 (18–44)	28 (19–45)	33 (20–45)	24 (18–32)	1.0 (1.0–1.1)	0.169	1.2* (1.1–1.3)	0.001

Median age of onset was determined using the actuarial method. Differences in age of onset were tested with a discrete-time logistic regression, with the sample limited to participants who developed the comorbid disorder, and with number of early phobia subtypes time-varying until age 13

*p < 0.005

### Impairment, suicidality, and serious mental illness

Respondents without childhood SP reported a mean (SE) of 1.05 (0.03) days out of role in the past 30 days, compared to 1.80 (0.19) days among respondents with one SP subtype and 3.53 (0.53) days among those with 4+ subtypes (*B* = 0.56 (total sample) and 0.49 (SP subsample), *p* < 0.001) (Table 5). Suicidality was also relatively common among those with childhood SP, with 31.8% of those with 4+ subtypes reporting lifetime suicidal ideation, compared to only 7.5% of those without childhood phobia and 18.8% of those with one SP subtype (OR = 1.6 (total sample) and 1.3 (SP subsample), *p* < 0.001). Furthermore, 36.8% of respondents with 4+ subtypes reported a 12-month SMI, compared to just 3.5% of those without childhood phobia and 12.7% of those with one subtype (OR = 2.1 (total sample) and 1.5 (SP subsample), *p* < 0.001). PAFs for suicidality and SMI ranged from 13.9% for suicidal ideation to 20.4% for suicide attempts (Additional file 1: Table S3).

**Table 5:** Days out of role, suicidality, and serious mental illness

Category	Subcategory	Number of subtypes					Test of linear effect (total sample)		Test of linear effect (SP cases only)	
		0	1	2	3	4+				
		Mean (SE)	Mean (SE)	Mean (SE)	Mean (SE)	Mean (SE)	*B* (SE)	*p* value	*B* (SE)	*p* value
Days out of role in the past 30 days		1.05 (0.03)	1.80 (0.19)	1.94 (0.17)	2.51 (0.30)	3.53 (0.53)	0.56* (0.06)	< 0.001	0.49* (0.15)	< 0.001
		% (SE)	% (SE)	% (SE)	% (SE)	% (SE)	OR (95% CI)	*p* value	OR (95% CI)	*p* value
Suicidality	Ideation	7.5 (0.1)	18.8 (0.8)	23.7 (1.3)	30.7 (2.0)	31.8 (2.4)	1.6* (1.6–1.7)	< 0.001	1.3* (1.2–1.3)	< 0.001
	Plan	2.3 (0.1)	6.7 (0.5)	9.5 (0.8)	13.6 (1.4)	14.1 (1.8)	1.7* (1.6–1.8)	< 0.001	1.3* (1.2–1.4)	< 0.001
	Attempt	2.0 (0.0)	6.5 (0.4)	8.4 (0.7)	13.4 (1.3)	16.8 (1.7)	1.7* (1.7–1.8)	< 0.001	1.4* (1.3–1.5)	< 0.001
Serious mental illness		3.5 (0.1)	12.7 (0.7)	20.9 (1.2)	27.8 (2.1)	36.8 (2.7)	2.1* (2.0–2.2)	< 0.001	1.5* (1.4–1.7)	< 0.001

*p < 0.005

### Sensitivity analyses

Associations of SP subtype number with prevalence, persistence, and severity of secondary comorbid disorders were generally consistent across age groups (Additional file 1: Table S4 and 5), suggesting that the associations described above are generally stable over the life course. Likewise, we found associations of SP subtype number with the lifetime prevalence of comorbid disorders and suicidality in participants with and without a current 12-month internalizing disorder (Additional file 1: Table S6-S10). Finally, the prevalence of specific phobia, comorbid disorders, and suicidality were all lower in low- or middle-income countries compared to high-income countries; however, the associations between number of SP subtypes and comorbidity or suicidality were similar regardless of country income level (Additional file 1: Table S11-16).

## Discussion

### Principal findings

In this study, we used a large cross-national sample to explore the associations of childhood generalized SP with the prevalence, persistence, and severity of other internalizing disorders. Among the 7.6% of participants reporting lifetime SP, most (78%) had already developed the disorder before age 13. Childhood SP was highly persistent, although severe disability and treatment-seeking for SP were uncommon. Many respondents with childhood SP reported multiple phobias (44%), with 8% even reporting four or more phobias. Childhood SP was strongly associated with prevalence, persistence, and severity of other internalizing disorders as well as with an early age of onset of these disorders. It was also associated with increased number of days out of role and SMI, as well as with suicidality. At a population level, 8.7–38.8% of all internalizing disorders and 13.9–20.4% of all suicidality or SMI were associated with childhood SP. Furthermore, associations persisted throughout the lifespan. Of particular importance, participants with generalized specific phobia had much worse outcomes than those with a single fear. For example, 17% of those with four or more phobias reported a suicide attempt, compared to only 7% of those with a single phobia.

SP is generally viewed as a relatively mild disorder that causes less disability than other mood or anxiety disorders [24]. However, our results suggest that childhood SP, particularly when generalized, is strongly associated with poor long-term outcomes in the internalizing domain. Caution is needed since these results are based on a retrospective cohort design and recall error could lead to bias. However, the stability of the results across the age range of this very large and diverse sample suggests that the pattern is worthy of future investigation.

The present study is a practical application of suggestions made in the Hierarchical Taxonomy Of Psychopathology (HiTOP) initiative, which aims to develop an empirically driven nosology of psychopathology [44]. One fundamental aspect of HiTOP is its reliance on quantitative, rather than dichotomous measures, to more closely approximate the true nature of psychopathology. Although we have examined a specific disorder, we have used a more quantitative approach than the strictly DSM approach by looking at the number of SP subtypes. Furthermore, we chose to focus on specific phobia because it tends to be the earliest manifestation of an internalizing liability, not because we think it is unique. The results suggest that it might be possible to identify at least some of the people with a strong internalizing liability in childhood even though their childhood disorders typically are not severe.

A similar approach could be applied to other psychopathology domains. For instance, the externalizing domain includes substance use disorders, which usually first appear in late adolescence, as well as disorders with childhood onset, such as attention deficit hyperactivity disorder (ADHD) [3]. For other domains, such as thought disorders, subclinical psychotic experiences or personality traits like suspiciousness might be relevant [45, 46]. Examination of personality traits might also help refine the early identification of persons with high internalizing or externalizing liabilities.

From the point of view of prevention, one might argue whether targeted interventions should be aimed at everyone with childhood SP or at a smaller or larger group. If resources are limited, targeting those with multiple phobias is reasonable, as the tendency to generalize fear appears to predict a particularly poor prognosis. For instance, reducing the risk for just this very small group (0.5%) to that of people without childhood SP could prevent 1.1–7.8% of other internalizing disorders and 3.5% of all suicide attempts. However, even participants with a single phobia had increased risks of unfavorable outcomes. The population attributable fraction for suicide attempts for all childhood SP, for instance, was 20.4%, showing that much greater benefits could be obtained by targeting this larger group. Furthermore, previous research has found that most children reporting a specific fear do not meet SP criteria [31]. It is currently unclear whether such fearfulness is harmless or a sign of an internalizing vulnerability, for instance when fearfulness is generalized.

As our study is observational, we cannot establish causality. We hypothesize that the associations are not directly causal, but that both childhood SP and later outcomes are expressions of a latent internalizing vulnerability. Although SP is a promising target in part because it is a relatively easy to treat disorder [47] that nevertheless often remains untreated, it is unclear whether treatment specifically for SP would have substantial effects on internalizing outcomes later in life. Exposure is a common element in cognitive behavioral therapy, so broader effects on the underlying vulnerability are plausible, and there is some evidence to suggest that treatment for phobia can lead to improvement in already-existing comorbid anxiety disorders [48]. Since even single-session behavioral therapy can be sufficient for SP [49], and since up to 20% of suicide attempts and 19% of SMI are attributable to childhood SP (assuming a causal relationship), it would be highly worthwhile to examine whether early treatment of specific phobia has a substantial effect on these other outcomes.

In contrast to most previous research regarding SP, which used participant samples from high-income Western countries, our sample included a diverse set of countries. We found that both SP and our main outcomes, comorbidity and suicidality, were less prevalent in low- and middle-income countries than in high-income countries. However, the associations between childhood (generalized) SP and these outcomes were remarkably similar across country income levels. This suggests that childhood generalized SP might be a globally useful and not culturally specific marker for at-risk children. However, treatment rates for childhood-onset SP are even lower in low- or middle-income countries (12.7%) than in high-income countries (26.8%), and it may be difficult to detect and provide early intervention to these children if resources are scarce. Ideally, to reach as many people as possible, any screening or intervention program for childhood generalized SP should also be feasible in countries with relatively few specialist mental health care providers.

### Strengths and limitations

The World Mental Health Surveys provide a unique opportunity to examine childhood SP and its relationship to other internalizing disorders in a very large sample from a diverse set of countries using a common protocol and instrument. However, there are also several important limitations. First, data are derived from a cross-sectional interview of adult participants, and age of onset is therefore estimated retrospectively. While the survey was designed using modern cognitive interviewing methods as a way to encourage active memory search and improve recall accuracy [50], some recall bias doubtlessly persists. Mild SP may have been forgotten, so our sample may not be fully representative of all children with SP. However, while some of our research questions could also be examined with existing longitudinal studies, no longitudinal studies with a sufficiently large sample size exists that could examine all the questions considered here. We also took several steps to investigate whether recall bias could explain our results. Firstly, we performed sensitivity analyses in the separate age groups and found broadly similar results in the youngest age group (18–34 years old), for whom recall bias should be least problematic, compared to the oldest age group (50+ years old). Presence of current psychopathology could also lead to recall bias. If respondents with current psychopathology are more likely to recall symptoms they suffered previously, including specific phobias, this could lead to a spurious association between childhood SP and comorbidity. However, we also found strong associations between number of childhood SP subtypes and lifetime comorbidity within the subgroup of respondents that did not report a 12-month internalizing disorder. Secondly, the observational design precludes causal inferences. Finally, the CIDI does not assess all phobias. Consequently, we may have underestimated the number of childhood SP subtypes.

## Conclusions

In conclusion, this study has shown that childhood generalized SP, as assessed in a retrospective survey, is strongly associated with poor outcomes in the internalizing domain of psychopathology throughout the life course. While our study cannot establish whether childhood SP is causally associated with these later outcomes or whether some other factor, such as an underlying internalizing vulnerability, explains the association, it clearly identifies children with generalized SP as a high-risk group. Respondents with childhood SP not only were more likely than other respondents to develop internalizing disorders, but developed them at an earlier age and had a more persistent and severe course, including more disability, suicidality, and serious mental illness. Although even respondents with a single childhood SP subtype had an elevated risk of poor outcomes, risk was much higher among respondents who reported multiple childhood SP subtypes. Children with generalized SP might therefore be an important target group for early intervention to reduce internalizing psychopathology across the lifespan.

## Additional file

Additional file 1: Supplemental tables providing additional information. (DOCX 52 kb)

## Abbreviations

ADHD: Attention deficit hyperactivity disorder
CI: Confidence interval
CIDI: Composite International Diagnostic Interview
DSM: Diagnostic and Statistical Manual of Mental Disorders
GAD: Generalized anxiety disorder
HiTOP: Hierarchical Taxonomy Of Psychopathology
IQR: Interquartile range
MDD: Major depressive disorder
OR: Odds ratio
PAF: Population attributable fraction
PTSD: Post-traumatic stress disorder
SCID: Structured Clinical Interview for DSM-IV
SD: Standard deviation
SDS: Sheehan Disability Scale
SE: Standard error
SMI: Serious mental illness
SP: Specific phobia
WMHS: World Mental Health Surveys

## References

[1] GBD 2015 Disease and Injury Incidence and Prevalence Collaborators (2016). Global, regional, and national incidence, prevalence, and years lived with disability for 310 diseases and injuries, 1990–2015: a systematic analysis for the Global Burden of Disease Study 2015. Lancet.

[2] The WHO World Mental Health Survey Consortium (2004). Prevalence, severity, and unmet need for treatment of mental disorders in the World Health Organization World Mental Health Surveys. JAMA.

[3] Kessler RC, Berglund PA, Demler O, Jin R, Merikangas KR, Walters EE (2005). Lifetime prevalence and age-of-onset distributions of DSM-IV disorders in the National Comorbidity Survey Replication. Arch Gen Psychiatry.

[4] Penninx BWJH, Nolen WA, Lamers F, Zitman FG, Smit JH, Spinhoven P (2011). Two-year course of depressive and anxiety disorders: results from the Netherlands Study of Depression and Anxiety (NESDA). J Affect Disord.

[5] Bruce SE, Yonkers KA, Otto MW, Eisen JL, Weisberg RB, Pagano M (2005). Influence of psychiatric comorbidity on recovery and recurrence in generalized anxiety disorder, social phobia, and panic disorder: a 12-year prospective study. Am J Psychiatry.

[6] Judd LL, Akiskal HS, Maser JD, Zeller PJ, Endicott J, Coryell W (1998). A prospective 12-year study of subsyndromal and syndromal depressive symptoms in unipolar major depressive disorders. Arch Gen Psychiatry.

[7] Turner EH, Matthews AM, Linardatos E, Tell RA, Rosenthal R (2008). Selective publication of antidepressant trials and its influence on apparent efficacy. N Engl J Med.

[8] Roest AM, de Jonge P, Williams CD, de Vries YA, Schoevers RA, Turner EH (2015). Reporting bias in clinical trials investigating the efficacy of second-generation antidepressants in the treatment of anxiety disorders: a report of 2 meta-analyses. JAMA Psychiatry..

[9] Huhn M, Tardy M, Spineli LM, Kissling W, Förstl H, Pitschel-Walz G (2014). Efficacy of pharmacotherapy and psychotherapy for adult psychiatric disorders: a systematic overview of meta-analyses. JAMA Psychiatry.

[10] Andrews G, Sanderson K, Corry J, Lapsley HM (2000). Using epidemiological data to model efficiency in reducing the burden of depression. J Ment Health Policy Econ.

[11] Chisholm D, Sanderson K, Ayuso-Mateos JL, Saxena S, C D, S K (2004). Reducing the global burden of depression: population-level analysis of intervention cost-effectiveness in 14 world regions. Br J Psychiatry.

[12] Kessler RC, Chiu WT, Demler O, Walters EE (2005). Prevalence, severity, and comorbidity of 12-month DSM-IV disorders in the National Comorbidity Survey Replication. Arch Gen Psychiatry.

[13] Ormel J, Raven D, van Oort F, Hartman CA, Reijneveld SA, Veenstra R (2015). Mental health in Dutch adolescents: a TRAILS report on prevalence, severity, age of onset, continuity and co-morbidity of DSM disorders. Psychol Med.

[14] Krueger RF (1999). The structure of common mental disorders. Arch Gen Psychiatry.

[15] de Jonge P, Wardenaar KJ, Lim CCW, Aguilar-Gaxiola S, Alonso J, Andrade LH (2018). The cross-national structure of mental disorders: results from the World Mental Health Surveys. Psychol Med.

[16] Kessler RC, Cox BJ, Green JG, Ormel J, McLaughlin KA, Merikangas KR (2011). The effects of latent variables in the development of comorbidity among common mental disorders. Depress Anxiety.

[17] Markon KE (2010). Modeling psychopathology structure: a symptom-level analysis of Axis I and II disorders. Psychol Med.

[18] Eaton NR, Keyes KM, Krueger RF, Balsis S, Skodol AE, Markon KE (2012). An invariant dimensional liability model of gender differences in mental disorder prevalence: evidence from a national sample. J Abnorm Psychol.

[19] Forbush KT, Watson D (2013). The structure of common and uncommon mental disorders. Psychol Med.

[20] Kessler RC, Ormel J, Petukhova M, McLaughlin KA, Green JG, Russo LJ (2011). Development of lifetime comorbidity in the World Health Organization world mental health surveys. Arch Gen Psychiatry.

[21] Wardenaar KJ, Lim CCW, Al-Hamzawi AO, Alonso J, Andrade LH, Benjet C (2017). The cross-national epidemiology of specific phobia in the World Mental Health Surveys. Psychol Med.

[22] de Jonge P, Scott KM, Stein DJ, Kessler RC (2018). Mental disorders around the world: facts and figures from the WHO World Mental Health Surveys.

[23] Comer JS, Blanco C, Hasin DS, Liu S-M, Grant BF, Turner B (2011). Health-related quality of life across the anxiety disorders. J Clin Psychiatry..

[24] Ormel J, Petukhova M, Chatterji S, Aguilar-Gaxiola S, Alonso J, Angermeyer MC (2008). Disability and treatment of specific mental and physical disorders across the world. Br J Psychiatry.

[25] Lieb R, Miche M, Gloster AT, Beesdo-Baum K, Meyer AH, Wittchen H-U (2016). Impact of specific phobia on the risk of onset of mental disorders: a 10-year prospective-longitudinal community study of adolescents and young adults. Depress Anxiety.

[26] Depla MFIA, ten Have ML, van Balkom AJLM, de Graaf R (2008). Specific fears and phobias in the general population: results from the Netherlands Mental Health Survey and Incidence Study (NEMESIS). Soc Psychiatry Psychiatr Epidemiol.

[27] Goodwin RD (2002). Anxiety disorders and the onset of depression among adults in the community. Psychol Med.

[28] Magee WJ, Eaton WW, Wittchen HU, McGonagle KA, Kessler RC (1996). Agoraphobia, simple phobia, and social phobia in the National Comorbidity Survey. Arch Gen Psychiatry.

[29] Park S, Sohn JH, Hong JP, Chang SM, Lee YM, Jeon HJ (2013). Prevalence, correlates, and comorbidities of four DSM-IV specific phobia subtypes: results from the Korean Epidemiological Catchment Area study. Psychiatry Res.

[30] Chou K-L (2009). Specific phobia in older adults: evidence from the National Epidemiologic Survey on Alcohol and Related Conditions. Am J Geriatr Psychiatry.

[31] Benjet C, Borges G, Stein DJ, Mendez E, Medina-Mora ME (2012). Epidemiology of fears and specific phobia in adolescence: results from the Mexican Adolescent Mental Health Survey. J Clin Psychiatry.

[32] Choy Y, Fyer AJ, Goodwin RD (2007). Specific phobia and comorbid depression: a closer look at the National Comorbidity Survey data. Compr Psychiatry.

[33] Stinson FS, D a D, Patricia Chou S, Smith S, Goldstein RB, June Ruan W (2007). The epidemiology of DSM-IV specific phobia in the USA: results from the National Epidemiologic Survey on Alcohol and Related Conditions. Psychol Med.

[34] Trumpf J, Margraf J, Vriends N, Meyer AH, Becker ES (2010). Predictors of specific phobia in young women: a prospective community study. J Anxiety Disord.

[35] Curtis GC, Magee WJ, Eaton WW, Wittchen HU, Kessler RC (1998). Specific fears and phobias. Epidemiology and classification. Br J Psychiatry.

[36] Burstein M, Georgiades K, He JP, Schmitz A, Feig E, Khazanov GK (2012). Specific phobia among U.S. adolescents: phenomenology and typology. Depress Anxiety.

[37] Heeringa SG, Wells JE, Hubbard F, Mneimneh Z, Chiu WT, Sampson N, Kessler RC, Üstün TB (2008). Sample designs and sampling procedures. The WHO World Mental Health Surveys: global perspectives on the epidemiology of mental disorders.

[38] Pennell B, Mneimneh Z, Bowers A, Chardoul S, Wells JE, Viana MC, Kessler RC, Üstün TB (2008). Implementation of the world mental health surveys. The WHO World Mental Health Surveys: global perspectives on the epidemiology of mental disorders.

[39] Kessler RC, Üstün BB (2004). The World Mental Health (WMH) Survey Initiative version of the World Health Organization (WHO) Composite International Diagnostic Interview (CIDI). Int J Methods Psychiatr Res.

[40] Haro JM, Arbabzadeh-Bouchez S, Brugha TS, De Girolamo G, Guyer ME, Jin R (2006). Concordance of the Composite International Diagnostic Interview version 3.0 (CIDI 3.0) with standardized clinical assessments in the WHO World Mental Health Surveys. Int J Methods Psychiatr Res.

[41] Sheehan DV, Harnett-Sheehan K, Raj BA (1996). The measurement of disability. Int Clin Psychopharmacol.

[42] Levinson D, Lakoma MD, Petukhova M, Schoenbaum M, Zaslavsky AM, Angermeyer M (2010). Associations of serious mental illness with earnings: results from the WHO World Mental Health surveys. Br J Psychiatry.

[43] Heeringa SG, Berglund PA, West BT, Mellipilán ER, Portier K (2015). Attributable fraction estimation from complex sample survey data. Ann Epidemiol.

[44] Kotov R, Krueger RF, Watson D, Achenbach TM, Althoff RR, Bagby RM (2017). The Hierarchical Taxonomy of Psychopathology (HiTOP): a dimensional alternative to traditional nosologies. J Abnorm Psychol.

[45] Kaymaz N, Drukker M, Lieb R, Wittchen H-U, Werbeloff N, Weiser M (2012). Do subthreshold psychotic experiences predict clinical outcomes in unselected non-help-seeking population-based samples? A systematic review and meta-analysis, enriched with new results. Psychol Med.

[46] Poulton R, Caspi A, Moffitt TE, Cannon M (2000). Children’s self-reported psychotic symptoms and adult schizophreniform disorder. Arch Gen Psychiatry.

[47] Choy Y, Fyer AJ, Lipsitz JD (2007). Treatment of specific phobia in adults. Clin Psychol Rev.

[48] Ryan SM, Strege MV, Oar EL, Ollendick TH (2017). One session treatment for specific phobias in children: comorbid anxiety disorders and treatment outcome. J Behav Ther Exp Psychiatry.

[49] Zlomke K, Davis TE (2008). One-session treatment of specific phobias: a detailed description and review of treatment efficacy. Behav Ther.

[50] Knäuper B, Cannell C, Schwarz N, Bruce M, Kessler RC (1999). Improving the accuracy of major depression age of onset reports in the US National Comorbidity Survey. Int J Methods Psychiatr Res.

