# Supplementary material for: Childhood generalized specific phobia as an early marker of internalizing psychopathology across the lifespan: results from the World Mental Health Surveys
Source: BMC Med. 2019 May 24;17:101. doi: 10.1186/s12916-019-1328-3 (PMC6533738; doi:10.1186/s12916-019-1328-3)
Supplement: Supplementary file 1 — Supplemental tables providing additional information. (DOCX 52 kb) [file 12916_2019_1328_MOESM1_ESM.docx]

**Additional file 1**

**Supplemental table 1.** WMH sample characteristics by World Bank income categories

**Supplemental table 2.** Population attributable fractions for each comorbid disorder, for any childhood specific phobia and for 1, 2, 3, or 4+ specific phobia subtypes

**Supplemental table 3.** Population attributable fractions for suicidality and serious mental illness, for any childhood specific phobia and for 1, 2, 3, or 4+ specific phobia subtypes

**Supplemental table 4.** Association between number of early-onset specific phobia subtypes and lifetime comorbidity in each age group, and test of interaction between age group and number of subtypes

**Supplemental table 5.** Association between number of early-onset specific phobia subtypes and suicidality or serious mental illness in each age group, and test of interaction between age group and number of subtypes

**Supplemental table 6:** Prevalence of any 12-month internalizing disorder, as a function of number of specific phobia subtypes

**Supplemental table 7:** Lifetime prevalence of comorbid internalizing disorders, as a function of number of specific phobia subtypes, in participants *without* current psychopathology

**Supplemental table 8:** Lifetime prevalence of comorbid internalizing disorders, as a function of number of specific phobia subtypes, in participants *with* current psychopathology

**Supplemental table 9:** Lifetime prevalence of suicidality according to number of specific phobia subtypes, among participants *without* current psychopathology

**Supplemental table 10:** Lifetime prevalence of suicidality according to number of specific phobia subtypes, among participants *with* current psychopathology

**Supplemental table 11.** Prevalence and characteristics of specific phobia, among participants from low or middle income countries

**Supplemental table 12.** Prevalence and characteristics of specific phobia, among participants from high income countries

**Supplemental table 13:** Prevalence of comorbid internalizing disorders, as a function of number of specific phobia subtypes, among participants from low or middle income countries

**Supplemental table 14:** Prevalence of comorbid internalizing disorders, as a function of number of specific phobia subtypes, among participants from high income countries

**Supplemental table 15:** Lifetime prevalence of suicidality according to number of specific phobia subtypes, among participants from low or middle income countries

**Supplemental table 16:** Lifetime prevalence of suicidality according to number of specific phobia subtypes, among participants from high income countries

| **Supplemental table 1.** WMH sample characteristics by World Bank income categories*^a^* | | | | | | | | |
| --- | --- | --- | --- | --- | --- | --- | --- | --- |
|  |  |  |  |  | **Sample size** | | |  |
| **Country by income category** | **Survey*^b^*** | **Sample characteristics*^c^*** | **Field dates** | **Age range** | **Part I** | **Part II** | **Response rate*^e^*** | |
| **I. Low and lower middle income countries** | | |  |  |  |  |  | |
| Colombia | NSMH | All urban areas of the country (approximately 73% of the total national population). | 2003 | 18-65 | 4,426 | 2,381 | 87.7 | |
| Iraq | IMHS | Nationally representative. | 2006-7 | 18-96 | 4,332 | 4,332 | 95.2 | |
| Nigeria | NSMHW | 21 of the 36 states in the country, representing 57% of the national population. The surveys were conducted in Yoruba, Igbo, Hausa and Efik languages. | 2002-4 | 18-100 | 6,752 | 2,143 | 79.3 | |
| PRC^f^  - Shenzhen^g^ | Shenzhen | Shenzhen metropolitan area. Included temporary residents as well as household residents. | 2005-7 | 18-88 | 7,132 | 2,475 | 80.0 | |
| Peru | EMSMP | Five urban areas of the country (approximately 38% of the total national population). | 2004-5 | 18-65 | 3,930 | 1,801 | 90.2 | |
| **TOTAL** |  |  |  |  | (26,572) | (13,132) | 84.7 | |
| **II. Upper-middle income countries** | | |  |  |  |  |  | |
| Brazil - São Paulo | São Paulo Megacity | São Paulo metropolitan area. | 2005-8 | 18-93 | 5,037 | 2,942 | 81.3 | |
| Bulgaria | NSHS | Nationally representative. | 2002-6 | 18-98 | 5,318 | 2,233 | 72.0 | |
| Colombia - Medellin^h^ | MMHHS | Medellin metropolitan area | 2011-12 | 19-65 | 3,261 | 1,673 | 97.2 | |
| Lebanon | LEBANON | Nationally representative. | 2002-3 | 18-94 | 2,857 | 1,031 | 70.0 | |
| Mexico | M-NCS | All urban areas of the country (approximately 75% of the total national population). | 2001-2 | 18-65 | 5,782 | 2,362 | 76.6 | |
| Romania | RMHS | Nationally representative. | 2005-6 | 18-96 | 2,357 | 2,357 | 70.9 | |
| **TOTAL** |  |  |  |  | (24,612) | (12,598) | 77.2 | |
| **III. High-income countries** | | |  |  |  |  |  | |
| Argentina | AMHES | Eight largest urban areas of the country (approximately 50% of the total national population) | 2015 | 18-98 | 3,927 | 2,116 | 77.3 | |
| Belgium | ESEMeD | Nationally representative. The sample was selected from a national register of Belgium residents. | 2001-2 | 18-95 | 2,419 | 1,043 | 50.6 | |
| France | ESEMeD | Nationally representative. The sample was selected from a national list of households with listed telephone numbers. | 2001-2 | 18-97 | 2,894 | 1,436 | 45.9 | |
| Germany | ESEMeD | Nationally representative. | 2002-3 | 19-95 | 3,555 | 1,323 | 57.8 | |
| Italy | ESEMeD | Nationally representative. The sample was selected from municipality resident registries. | 2001-2 | 18-100 | 4,712 | 1,779 | 71.3 | |
| Japan | WMHJ 2002-2006 | Eleven metropolitan areas. | 2002-6 | 20-98 | 4,129 | 1,682 | 55.1 | |
| Netherlands | ESEMeD | Nationally representative. The sample was selected from municipal postal registries. | 2002-3 | 18-95 | 2,372 | 1,094 | 56.4 | |
| New Zealand^g^ | NZMHS | Nationally representative. | 2004-5 | 18-98 | 12,790 | 7,312 | 73.3 | |
| N. Ireland | NISHS | Nationally representative. | 2005-8 | 18-97 | 4,340 | 1,986 | 68.4 | |
| Poland | EZOP | Nationally representative | 2010-11 | 18-65 | 10,081 | 4,000 | 50.4 | |
| Portugal | NMHS | Nationally representative. | 2008-9 | 18-81 | 3,849 | 2,060 | 57.3 | |
| Spain | ESEMeD | Nationally representative. | 2001-2 | 18-98 | 5,473 | 2,121 | 78.6 | |
| Spain - Murcia | PEGASUS- Murcia | Murcia region. Regionally representative. | 2010-12 | 18-96 | 2,621 | 1,459 | 67.4 | |
| United States | NCS-R | Nationally representative. | 2001-3 | 18-99 | 9,282 | 5,692 | 70.9 | |
| **TOTAL** |  |  |  |  | (72,444) | (35,103) | 63.0 | |
| **IV. TOTAL** |  |  |  |  | (123,628) | (60,833) | 69.3 | |
|  | | | | | | | | |

*^a^* The World Bank (2012) Data. Accessed May 12, 2012 at: <http://data.worldbank.org/country>. Some of the WMH countries have moved into new income categories since the surveys were conducted. The income groupings above reflect the status of each country at the time of data collection. The current income category of each country is available at the preceding URL.

*^b^*NSMH (The Colombian National Study of Mental Health); IMHS (Iraq Mental Health Survey); NSMHW (The Nigerian Survey of Mental Health and Wellbeing); EMSMP (La Encuesta Mundial de Salud Mental en el Peru); NSHS (Bulgaria National Survey of Health and Stress); MMHHS (Medellín Mental Health Household Study); LEBANON (Lebanese Evaluation of the Burden of Ailments and Needs of the Nation); M-NCS (The Mexico National Comorbidity Survey); RMHS (Romania Mental Health Survey); AMHES (Argentina Mental Health Epidemiologic Survey); ESEMeD (The European Study Of The Epidemiology Of Mental Disorders); WMHJ2002-2006 (World Mental Health Japan Survey); NZMHS (New Zealand Mental Health Survey); NISHS (Northern Ireland Study of Health and Stress); EZOP (Epidemiology of Mental Disorders and Access to Care Survey); NMHS (Portugal National Mental Health Survey); PEGASUS-Murcia (Psychiatric Enquiry to General Population in Southeast Spain-Murcia);NCS-R (The US National Comorbidity Survey Replication).

*^c^* Most WMH surveys are based on stratified multistage clustered area probability household samples in which samples of areas equivalent to counties or municipalities in the US were selected in the first stage followed by one or more subsequent stages of geographic sampling (e.g., towns within counties, blocks within towns, households within blocks) to arrive at a sample of households, in each of which a listing of household members was created and one or two people were selected from this listing to be interviewed. No substitution was allowed when the originally sampled household resident could not be interviewed. These household samples were selected from Census area data in all countries other than France (where telephone directories were used to select households) and the Netherlands (where postal registries were used to select households). Several WMH surveys (Belgium, Germany, Italy, Poland, Spain-Murcia) used municipal, country resident or universal health-care registries to select respondents without listing households. The Japanese sample is the only totally un-clustered sample, with households randomly selected in each of the 11 metropolitan areas and one random respondent selected in each sample household. 15 of the 25 surveys are based on nationally representative household samples.

*^d^* Argentina, Brazil, Colombia-Medellin, Iraq, Japan, New Zealand, Northern Ireland, PRC - Shenzhen, Romania and Spain-Murcia did not have an age restricted Part 2 sample. All other countries, with the exception of Nigeria (which was age restricted to ≤ 39) were age restricted to ≤ 44.

*^e^* The response rate is calculated as the ratio of the number of households in which an interview was completed to the number of households originally sampled, excluding from the denominator households known not to be eligible either because of being vacant at the time of initial contact or because the residents were unable to speak the designated languages of the survey. The weighted average response rate is 69.3%.

*^f^* People’s Republic of China

*^g^* For the purposes of cross-national comparisons we limit the sample to those 18+.

*^h^*Colombia moved from the "lower and lower-middle income" to the "upper-middle income" category between 2003 (when the Colombian National Study of Mental Health was conducted) and 2010 (when the Medellin Mental Health Household Study was conducted), hence Colombia's appearance in both income categories. For more information, please see footnote *a*.

**Supplemental table 2.** Population attributable fractions for each comorbid disorder, for any childhood specific phobia and for 1, 2, 3, or 4+ specific phobia subtypes

| **Disorder** | **Any early-onset SP** | **Number of subtypes** | | | |
| --- | --- | --- | --- | --- | --- |
|  |  | **1** | **2** | **3** | **4** |
|  | **% (95% CI)** | **% (95% CI)** | **% (95% CI)** | **% (95% CI)** | **% (95% CI)** |
| Agoraphobia | 38.8 (34.7-42.7) | 10.8 (7.6-13.8) | 10.9 (8.8-13.0) | 9.4 (6.5-12.2) | 7.8 (5.8-9.6) |
| Generalized anxiety disorder | 13.3 (11.6-15.0) | 4.8 (3.7-5.9) | 4.1 (3.2-4.9) | 2.5 (1.7-3.2) | 2.0 (1.3-2.7) |
| Panic disorder | 21.7 (18.8-24.5) | 9.0 (6.5-11.5) | 5.9 (4.3-7.4) | 3.3 (1.4-5.1) | 3.5 (2.3-4.8) |
| Post-traumatic stress disorder | 16.9 (14.6-19.2) | 7.0 (4.8-9.2) | 5.0 (3.4-6.6) | 2.1 (0.3-3.9) | 2.9 (2.1-3.6) |
| Separation anxiety disorder | 18.2 (15.5-20.7) | 7.5 (5.5-9.3) | 5.4 (4.0-6.8) | 2.8 (1.4-4.3) | 2.4 (1.3-3.5) |
| Social phobia | 23.1 (20.8-25.3) | 8.8 (7.4-10.2) | 6.1 (4.4-7.7) | 4.3 (3.3-5.2) | 3.9 (3.1-4.7) |
| **Any anxiety disorder** | 14.5 (13.4-15.7) | 6.3 (5.6-7.1) | 3.9 (3.1-4.7) | 2.3 (1.8-2.8) | 2.0 (1.7-2.3) |
| Major depression/dysthymia | 8.7 (7.8-9.6) | 4.2 (3.1-5.2) | 2.0 (1.1-2.9) | 1.5 (1.1-1.8) | 1.1 (0.7-1.4) |
| Bipolar disorder | 17.3 (13.4-21.0) | 5.1 (3.0-7.2) | 5.9 (4.5-7.2) | 2.9 (1.6-4.1) | 3.5 (2.4-4.6) |
| **Any mood disorder** | 9.9 (9.1-10.7) | 4.3 (3.5-5.0) | 2.6 (1.8-3.4) | 1.7 (1.4-1.9) | 1.4 (1.1-1.7) |
| Bulimia nervosa | 21.9 (15.7-27.6) | 6.3 (2.6-9.8) | 6.1 (3.1-9.1) | 5.8 (3.1-8.5) | 3.6 (1.6-5.6) |
| Binge eating disorder | 11.8 (2.2-20.5) | 4.8 (-3.6-12.5) | 3.2 (1.1-5.3) | 1.5 (-0.5-3.5) | 2.3 (0.4-4.1) |
| **Any eating disorder** | 14.8 (9.0-20.2) | 5.1 (-0.2-10.0) | 4.1 (2.5-5.7) | 2.9 (1.6-4.2) | 2.7 (1.3-4.1) |
| **Any internalizing disorder** | 10.2 (9.5-10.9) | 4.7 (4.0-5.4) | 2.6 (1.9-3.4) | 1.6 (1.3-1.9) | 1.3 (1.1-1.5) |
| Exactly 1 INT disorder | 5.2 (4.4-6.0) | 3.3 (2.2-4.3) | 1.2 (0.3-2.1) | 0.4 (-0.0-0.9) | 0.3 (0.1-0.5) |
| Exactly 2 INT disorders | 13.2 (11.7-14.6) | 6.2 (5.0-7.3) | 3.4 (2.6-4.2) | 2.4 (1.8-3.0) | 1.3 (0.5-2.0) |
| Exactly 3 INT disorders | 22.3 (19.2-25.2) | 7.6 (4.8-10.3) | 5.8 (3.7-7.9) | 5.0 (2.8-7.1) | 3.9 (2.7-5.1) |
| 4+ INT disorders | 39.4 (33.3-44.9) | 11.8 (9.3-14.2) | 12.3 (9.7-14.8) | 6.6 (2.3-10.8) | 8.7 (6.1-11.2) |

**Supplemental table 3.** Population attributable fractions for suicidality and serious mental illness, for any childhood specific phobia and for 1, 2, 3, or 4+ specific phobia subtypes

| **Outcome** | **Sub-outcome** | **Any early-onset SP** | **Number of subtypes** | | | |
| --- | --- | --- | --- | --- | --- | --- |
|  |  |  | **1** | **2** | **3** | **4** |
|  |  | **% (95% CI)** | **% (95% CI)** | **% (95% CI)** | **% (95% CI)** | **% (95% CI)** |
| **Suicidality** | Ideation | 13.9 (11.9-15.8) | 6.4 (5.2-7.5) | 3.7 (2.7-4.8) | 2.2 (1.1-3.2) | 1.7 (1.0-2.5) |
|  | Plan | 18.9 (15.5-22.1) | 7.1 (5.4-8.8) | 5.2 (3.5-7.0) | 3.5 (2.0-4.9) | 3.3 (2.3-4.4) |
|  | Attempt | 20.4 (16.3-24.2) | 8.7 (6.5-10.8) | 5.3 (3.7-6.8) | 3.2 (0.5-5.8) | 3.5 (1.6-5.4) |
| **Serious mental illness** |  | 19.1 (16.7-21.5) | 6.9 (5.7-8.1) | 5.4 (4.2-6.7) | 3.4 (2.7-4.1) | 3.4 (2.5-4.2) |

**Supplemental table 4.** Association between number of early-onset specific phobia subtypes and lifetime comorbidity in each age group, and test of interaction between age group and number of subtypes

| **Disorder** | **In whole sample** | | | | | | **In cases only** | | | | | |
| --- | --- | --- | --- | --- | --- | --- | --- | --- | --- | --- | --- | --- |
|  | **Linear effect of number of subtypes within age groups** | | | **Interaction between age group and number of subtypes** | | | **Linear effect of number of subtypes within age groups** | | | **Interaction between age group and number of subtypes** | | |
|  | **18 - 34 years old** | **35 - 49 years old** | **50+ years old** |  |  |  | **18 - 34 years old** | **35 - 49 years old** | **50+ years old** |  |  |  |
|  | **OR**  **(95% CI)** | **OR**  **(95% CI)** | **OR**  **(95% CI)** | **χ2** | **p** | **DF** | **OR**  **(95% CI)** | **OR**  **(95% CI)** | **OR**  **(95% CI)** | **χ2** | **p** | **DF** |
| Agoraphobia | 2.4*  (2.2-2.7) | 2.6*  (2.4-2.8) | 2.8*  (2.5-3.1) | 7.0 | 0.030 | 2 | 1.9*  (1.5-2.3) | 1.7*  (1.4-2.0) | 1.5*  (1.3-1.9) | 0.7 | 0.699 | 2 |
| Generalized anxiety disorder | 1.7*  (1.5-1.8) | 1.8*  (1.7-1.9) | 2.0*  (1.9-2.2) | 8.7 | 0.013 | 2 | 1.4*  (1.1-1.7) | 1.4*  (1.2-1.6) | 1.5*  (1.3-1.7) | 0.1 | 0.972 | 2 |
| Panic disorder | 2.0*  (1.8-2.2) | 2.1*  (1.9-2.3) | 2.0*  (1.8-2.2) | 0.0 | 0.988 | 2 | 1.6*  (1.3-1.9) | 1.5*  (1.3-1.8) | 1.3*  (1.1-1.6) | 0.7 | 0.696 | 2 |
| Post-traumatic  stress disorder | 1.8*  (1.7-2.0) | 1.9*  (1.8-2.1) | 2.1*  (1.9-2.3) | 3.1 | 0.215 | 2 | 1.5*  (1.3-1.8) | 1.3*  (1.2-1.5) | 1.4*  (1.2-1.6) | 3.5 | 0.173 | 2 |
| Separation anxiety disorder | 1.8*  (1.7-1.9) | 1.8*  (1.7-2.0) | 2.1*  (1.8-2.4) | 4.2 | 0.124 | 2 | 1.5*  (1.3-1.7) | 1.3  (1.1-1.5) | 1.4*  (1.1-1.8) | 3.7 | 0.158 | 2 |
| Social phobia | 2.1*  (1.9-2.2) | 2.3*  (2.1-2.4) | 2.5*  (2.2-2.7) | 10.3 | 0.006 | 2 | 1.5*  (1.3-1.7) | 1.4*  (1.2-1.6) | 1.6*  (1.4-1.9) | 0.8 | 0.663 | 2 |
| **Any anxiety disorder** | **2.2***  **(2.1-2.4)** | **2.3***  **(2.1-2.5)** | **2.8***  **(2.5-3.1)** | **16.1*** | **<.001** | **2** | **1.7***  **(1.5-2.0)** | **1.4***  **(1.3-1.6)** | **1.7***  **(1.5-2.0)** | **4.9** | **0.088** | **2** |
| Major depression/ dysthymia | 1.6*  (1.5-1.7) | 1.6*  (1.5-1.7) | 1.8*  (1.7-1.9) | 6.1 | 0.047 | 2 | 1.2*  (1.1-1.4) | 1.3*  (1.1-1.4) | 1.3*  (1.1-1.5) | 0.2 | 0.922 | 2 |
| Bipolar disorder | 1.8*  (1.6-1.9) | 1.9*  (1.8-2.1) | 2.0*  (1.8-2.2) | 5.3 | 0.069 | 2 | 1.6*  (1.4-1.9) | 1.3*  (1.1-1.5) | 1.4*  (1.1-1.7) | 4.6 | 0.098 | 2 |
| **Any mood disorder** | **1.8***  **(1.7-1.9)** | **1.9***  **(1.7-2.0)** | **2.0***  **(1.8-2.1)** | **2.2** | **0.331** | **2** | **1.5***  **(1.3-1.6)** | **1.4***  **(1.2-1.6)** | **1.4***  **(1.2-1.6)** | **1.1** | **0.569** | **2** |
| Bulimia nervosa | 1.8*  (1.6-2.1) | 2.0*  (1.7-2.4) | 2.1*  (1.7-2.6) | 1.4 | 0.491 | 2 | 1.8*  (1.3-2.4) | 1.6  (1.2-2.3) | 1.5  (1.1-2.1) | 0.9 | 0.645 | 2 |
| Binge eating disorder | 1.6*  (1.4-1.8) | 1.7*  (1.5-1.9) | 1.6*  (1.3-1.9) | 0.2 | 0.898 | 2 | 1.0  (0.7-1.3) | 1.3  (0.9-1.8) | 0.7  (0.4-1.3) | 3.8 | 0.147 | 2 |
| **Any eating disorder** | **1.7***  **(1.5-1.9)** | **1.8***  **(1.6-2.0)** | **1.7***  **(1.5-2.0)** | **0.4** | **0.834** | **2** | **1.3**  **(1.0-1.6)** | **1.4**  **(1.1-1.9)** | **0.9**  **(0.6-1.4)** | **2.9** | **0.231** | **2** |
| **Any internalizing disorder** | **2.2***  **(2.1-2.4)** | **2.3***  **(2.1-2.6)** | **2.9***  **(2.6-3.2)** | **14.1*** | **<.001** | **2** | **1.7***  **(1.5-1.9)** | **1.5***  **(1.3-1.8)** | **1.8***  **(1.5-2.2)** | **1.4** | **0.502** | **2** |
| Exactly 1 INT disorder | 1.3*  (1.3-1.4) | 1.2*  (1.2-1.3) | 1.5*  (1.4-1.6) | 13.8* | 0.001 | 2 | 1.1  (0.9-1.2) | 0.9  (0.8-1.1) | 1.1  (0.9-1.3) | 0.9 | 0.629 | 2 |
| Exactly 2 INT disorders | 1.7*  (1.6-1.8) | 1.6*  (1.5-1.8) | 1.8*  (1.6-1.9) | 2.3 | 0.309 | 2 | 1.3*  (1.1-1.5) | 1.1  (1.0-1.3) | 1.2  (1.0-1.4) | 2.1 | 0.342 | 2 |
| Exactly 3 INT disorders | 1.7*  (1.6-1.9) | 2.0*  (1.8-2.1) | 2.5*  (2.2-2.7) | 26.3* | <.001 | 2 | 1.3*  (1.1-1.5) | 1.3*  (1.1-1.6) | 1.6*  (1.3-1.9) | 3.2 | 0.197 | 2 |
| 4+ INT disorders | 2.5*  (2.3-2.8) | 2.7*  (2.4-2.9) | 2.6*  (2.3-2.9) | 0.3 | 0.852 | 2 | 2.0*  (1.6-2.4) | 1.7*  (1.4-2.0) | 1.5*  (1.2-1.8) | 3.4 | 0.179 | 2 |

* p < 0.005

**Supplemental table 5.** Association between number of early-onset specific phobia subtypes and suicidality or serious mental illness in each age group, and test of interaction between age group and number of subtypes

| **Category** | **In whole sample** | | | | | | **In cases only** | | | | | |
| --- | --- | --- | --- | --- | --- | --- | --- | --- | --- | --- | --- | --- |
|  | **Linear effect of number of subtypes within age groups** | | | **Interaction between age group and number of subtypes** | | | **Linear effect of number of subtypes within age groups** | | | **Interaction between age group and number of subtypes** | | |
|  | **18 - 34 years old** | **35 - 49 years old** | **50+ years old** |  |  |  | **18 - 34 years old** | **35 - 49 years old** | **50+ years old** |  |  |  |
|  | **OR**  **(95% CI)** | **OR**  **(95% CI)** | **OR**  **(95% CI)** | **χ2** | **p** | **DF** | **OR**  **(95% CI)** | **OR**  **(95% CI)** | **OR**  **(95% CI)** | **χ2** | **p** | **DF** |
| **Suicidal ideation** | 1.6*  (1.5-1.7) | 1.7*  (1.6-1.8) | 1.7*  (1.6-1.8) | 1.8 | 0.415 | 2 | 1.3*  (1.1-1.4) | 1.2*  (1.1-1.4) | 1.3*  (1.2-1.5) | 1.3 | 0.509 | 2 |
| **Suicidal plans** | 1.7*  (1.5-1.8) | 1.7*  (1.6-1.8) | 1.7*  (1.6-1.9) | 0.4 | 0.830 | 2 | 1.4*  (1.2-1.6) | 1.1  (1.0-1.3) | 1.4*  (1.2-1.8) | 6.3 | 0.043 | 2 |
| **Suicide attempt** | 1.7*  (1.6-1.8) | 1.8*  (1.6-1.9) | 1.8*  (1.7-2.0) | 0.5 | 0.760 | 2 | 1.5*  (1.3-1.7) | 1.2  (1.0-1.4) | 1.6*  (1.3-1.9) | 7.2 | 0.028 | 2 |
| **Serious mental illness** | 2.1*  (1.9-2.2) | 2.0*  (1.9-2.2) | 2.3*  (2.1-2.4) | 1.8 | 0.410 | 2 | 1.7*  (1.5-1.9) | 1.4*  (1.2-1.6) | 1.5*  (1.3-1.8) | 2.7 | 0.254 | 2 |

* p < 0.005

**Supplemental table 6.** Prevalence of any 12-month internalizing disorder, as a function of number of specific phobia subtypes

|  | **Number of subtypes** | | | | | **Test of linear effect**  **(total sample)** | | **Test of linear effect**  **(SP cases only)** | |
| --- | --- | --- | --- | --- | --- | --- | --- | --- | --- |
|  | **0** | **1** | **2** | **3** | **4+** |  |  |  |  |
|  | *% (SE)* | *% (SE)* | *% (SE)* | *% (SE)* | *% (SE)* | *OR (95% CI)* | *p-value* | *OR (95% CI)* | *p-value* |
| Any 12-month internalizing disorder | 8.7 (0.1) | 27.3 (1.0) | 40.4 (1.6) | 53.3 (2.4) | 59.8 (2.8) | 2.3* (2.2-2.4) | <.001 | 1.6* (1.5-1.7) | <.001 |

**Supplemental table 7.** Prevalence of comorbid internalizing disorders, as a function of number of specific phobia subtypes, in participants *without* current psychopathology

| **Comorbid disorder** | **Number of subtypes** | | | | | **Test of linear effect**  **(total sample)** | | **Test of linear effect**  **(SP cases only)** | |
| --- | --- | --- | --- | --- | --- | --- | --- | --- | --- |
|  | **0** | **1** | **2** | **3** | **4+** |  |  |  |  |
|  | *% (SE)* | *% (SE)* | *% (SE)* | *% (SE)* | *% (SE)* | *OR (95% CI)* | *p-value* | *OR (95% CI)* | *p-value* |
| Agoraphobia | 0.2 (0.0) | 1.2 (0.2) | 2.1 (0.7) | 6.8 (1.9) | 2.8 (1.1) | 2.4* (2.1-2.7) | <.001 | 1.8* (1.4-2.3) | <.001 |
| Generalized anxiety disorder | 1.5 (0.1) | 3.7 (0.4) | 4.7 (0.8) | 10.1 (2.0) | 5.9 (1.7) | 1.7* (1.5-1.9) | <.001 | 1.4* (1.2-1.6) | <.001 |
| Panic disorder | 0.5 (0.0) | 2.1 (0.4) | 2.0 (0.5) | 4.0 (1.3) | 3.8 (1.3) | 1.9* (1.7-2.2) | <.001 | 1.3 (1.0-1.7) | 0.073 |
| Post-traumatic stress disorder | 1.3 (0.1) | 3.3 (0.5) | 5.2 (1.0) | 8.8 (1.9) | 7.9 (2.5) | 1.8* (1.6-2.0) | <.001 | 1.5* (1.2-1.8) | <.001 |
| Separation anxiety disorder | 2.3 (0.1) | 7.4 (0.9) | 11.3 (1.9) | 18.1 (3.4) | 10.8 (2.6) | 1.8* (1.7-2.0) | <.001 | 1.3* (1.1-1.5) | 0.004 |
| Social phobia | 1.2 (0.1) | 5.2 (0.6) | 6.7 (1.1) | 10.0 (1.6) | 11.0 (3.2) | 2.0* (1.8-2.1) | <.001 | 1.4* (1.1-1.6) | <.001 |
| **Any anxiety disorder** | **5.2 (0.1)** | **16.3 (1.0)** | **22.2 (1.9)** | **36.3 (3.4)** | **28.2 (4.0)** | **2.0* (1.9-2.1)** | **<.001** | **1.4* (1.3-1.6)** | **<.001** |
| Major depression/dysthymia | 6.1 (0.1) | 12.5 (0.8) | 16.2 (1.6) | 20.5 (2.7) | 15.1 (2.8) | 1.5* (1.4-1.6) | <.001 | 1.2* (1.1-1.4) | <.001 |
| Bipolar disorder | 0.7 (0.0) | 2.2 (0.4) | 3.2 (0.8) | 4.5 (1.4) | 5.7 (1.9) | 1.8* (1.6-2.0) | <.001 | 1.3* (1.0-1.6) | 0.039 |
| **Any mood disorder** | **6.7 (0.1)** | **14.3 (0.8)** | **19.1 (1.7)** | **24.6 (2.7)** | **20.6 (3.3)** | **1.6* (1.5-1.7)** | **<.001** | **1.3* (1.1-1.4)** | **<.001** |
| Bulimia nervosa | 0.2 (0.0) | 0.4 (0.1) | 0.8 (0.4) | 2.7 (1.3) | 1.1 (0.8) | 1.8* (1.5-2.3) | <.001 | 2.0* (1.4-3.0) | <.001 |
| Binge eating disorder | 0.6 (0.1) | 1.6 (0.6) | 1.2 (0.5) | 4.2 (2.0) | 1.1 (0.8) | 1.5* (1.2-1.9) | <.001 | 1.2 (0.7-2.0) | 0.484 |
| **Any eating disorder** | **0.9 (0.1)** | **2.0 (0.7)** | **2.0 (0.6)** | **6.0 (2.3)** | **1.7 (1.0)** | **1.6* (1.3-1.9)** | **<.001** | **1.4 (0.9-2.0)** | **0.106** |
| **Any internalizing disorder** | **10.4 (0.2)** | **26.1 (1.2)** | **34.7 (2.2)** | **47.7 (3.4)** | **39.2 (4.4)** | **1.9* (1.8-2.0)** | **<.001** | **1.4* (1.2-1.5)** | **<.001** |
| Exactly 1 INT disorder | 8.3 (0.1) | 19.0 (1.1) | 24.5 (1.9) | 26.7 (3.0) | 26.0 (4.0) | 1.6* (1.5-1.7) | <.001 | 1.2* (1.1-1.3) | 0.005 |
| Exactly 2 INT disorders | 1.7 (0.1) | 5.0 (0.5) | 7.7 (1.2) | 13.3 (2.4) | 8.5 (1.8) | 1.8* (1.7-2.0) | <.001 | 1.4* (1.2-1.6) | <.001 |
| Exactly 3 INT disorders | 0.3 (0.0) | 1.7 (0.3) | 1.8 (0.5) | 4.5 (1.2) | 3.1 (1.3) | 2.1* (1.8-2.3) | <.001 | 1.4* (1.1-1.7) | 0.01 |
| 4+ INT disorders | 0.1 (0.0) | 0.4 (0.1) | 0.8 (0.3) | 3.1 (1.3) | 1.7 (1.0) | 2.5* (2.1-2.9) | <.001 | 2.0* (1.4-2.8) | <.001 |

**Supplemental table 8.** Prevalence of comorbid internalizing disorders, as a function of number of specific phobia subtypes, in participants *with* current psychopathology

| **Comorbid disorder** | **Number of subtypes** | | | | | **Test of linear effect**  **(total sample)** | | **Test of linear effect**  **(SP cases only)** | |
| --- | --- | --- | --- | --- | --- | --- | --- | --- | --- |
|  | **0** | **1** | **2** | **3** | **4+** |  |  |  |  |
|  | *% (SE)* | *% (SE)* | *% (SE)* | *% (SE)* | *% (SE)* | *OR (95% CI)* | *p-value* | *OR (95% CI)* | *p-value* |
| Agoraphobia | 6.3 (0.4) | 15.3 (1.3) | 20.4 (1.7) | 26.7 (3.1) | 33.6 (3.3) | 1.7* (1.6-1.8) | <.001 | 1.4* (1.2-1.5) | <.001 |
| Generalized anxiety disorder | 24.4 (0.6) | 25.8 (1.6) | 28.3 (2.1) | 30.6 (2.6) | 31.7 (3.6) | 1.1* (1.1-1.2) | <.001 | 1.1 (1.0-1.3) | 0.079 |
| Panic disorder | 10.7 (0.5) | 14.6 (1.3) | 18.7 (1.5) | 20.1 (2.5) | 23.9 (2.9) | 1.3* (1.2-1.4) | <.001 | 1.2* (1.1-1.4) | <.001 |
| Post-traumatic stress disorder | 19.1 (0.6) | 23.6 (1.6) | 29.8 (2.2) | 26.8 (2.7) | 31.5 (3.3) | 1.2* (1.2-1.3) | <.001 | 1.1* (1.0-1.3) | 0.01 |
| Separation anxiety disorder | 18.9 (0.7) | 26.3 (2.2) | 31.4 (2.6) | 35.2 (3.7) | 39.1 (3.9) | 1.3* (1.2-1.4) | <.001 | 1.2* (1.1-1.4) | 0.003 |
| Social phobia | 24.6 (0.6) | 40.7 (1.9) | 47.5 (2.2) | 50.0 (3.3) | 56.6 (3.6) | 1.5* (1.4-1.5) | <.001 | 1.2* (1.1-1.3) | <.001 |
| **Any anxiety disorder** | **66.2 (0.7)** | **79.7 (1.8)** | **83.5 (1.7)** | **85.4 (2.4)** | **91.6 (2.3)** | **1.5* (1.4-1.7)** | **<.001** | **1.3* (1.1-1.5)** | **0.002** |
| Major depression/dysthymia | 56.8 (0.8) | 54.9 (2.0) | 50.6 (2.3) | 49.9 (3.4) | 46.9 (3.5) | 0.9* (0.9-1.0) | 0.007 | 0.9 (0.8-1.0) | 0.121 |
| Bipolar disorder | 12.7 (0.5) | 14.1 (1.3) | 19.7 (1.8) | 19.7 (2.5) | 25.0 (2.8) | 1.2* (1.1-1.3) | <.001 | 1.3* (1.1-1.4) | <.001 |
| **Any mood disorder** | **68.1 (0.7)** | **67.4 (1.9)** | **69.0 (2.1)** | **68.1 (3.2)** | **71.2 (3.2)** | **1.0 (1.0-1.1)** | **0.139** | **1.1 (1.0-1.2)** | **0.181** |
| Bulimia nervosa | 4.3 (0.4) | 5.5 (1.0) | 7.1 (1.5) | 10.4 (2.7) | 9.1 (2.5) | 1.3* (1.2-1.5) | <.001 | 1.3* (1.1-1.7) | 0.009 |
| Binge eating disorder | 8.3 (0.5) | 14.8 (2.2) | 10.6 (1.7) | 8.6 (2.5) | 9.8 (2.7) | 1.1* (1.0-1.2) | 0.021 | 0.8 (0.6-1.1) | 0.144 |
| **Any eating disorder** | **11.7 (0.6)** | **18.9 (2.2)** | **16.5 (2.0)** | **17.8 (3.1)** | **17.8 (3.4)** | **1.2* (1.1-1.3)** | **<.001** | **1.0 (0.8-1.2)** | **0.823** |
| **Any internalizing disorder** | **100.0** | **100.0** | **100.0** | **100.0** | **100.0** | **-** |  | **-** |  |
| Exactly 1 INT disorder | 54.3 (0.7) | 37.3 (2.0) | 32.3 (2.3) | 26.8 (2.9) | 18.7 (3.0) | 0.6* (0.6-0.7) | <.001 | 0.8* (0.7-0.9) | <.001 |
| Exactly 2 INT disorders | 27.6 (0.6) | 31.3 (1.9) | 26.3 (2.1) | 28.9 (2.9) | 27.2 (3.2) | 1.0 (0.9-1.1) | 0.861 | 0.9 (0.8-1.0) | 0.085 |
| Exactly 3 INT disorders | 12.2 (0.5) | 17.2 (1.4) | 20.2 (1.8) | 20.1 (2.6) | 22.8 (2.8) | 1.2* (1.2-1.3) | <.001 | 1.1 (1.0-1.2) | 0.084 |
| 4+ INT disorders | 6.0 (0.4) | 14.2 (1.2) | 21.3 (1.9) | 24.2 (2.8) | 31.3 (3.7) | 1.7* (1.6-1.8) | <.001 | 1.4* (1.2-1.6) | <.001 |

**Supplemental table 9.** Lifetime prevalence of suicidality according to number of specific phobia subtypes, among participants *without* current psychopathology

| **Category** | **Subcategory** | **Number of subtypes** | | | | | **Test of linear effect**  **(total sample)** | | **Test of linear effect**  **(SP cases only)** | |
| --- | --- | --- | --- | --- | --- | --- | --- | --- | --- | --- |
|  |  | **0** | **1** | **2** | **3** | **4+** |  |  |  |  |
|  | | *% (SE)* | *% (SE)* | *% (SE)* | *% (SE)* | *% (SE)* | *OR (95% CI)* | *p-value* | *OR (95% CI)* | *p-value* |
| **Suicidality** | *Ideation* | 5.8 (0.1) | 12.6 (0.7) | 14.8 (1.4) | 19.6 (2.2) | 14.7 (2.5) | 1.5* (1.4-1.6) | <.001 | 1.1* (1.0-1.3) | 0.015 |
|  | *Plan* | 1.5 (0.0) | 4.3 (0.5) | 4.7 (0.8) | 5.7 (1.1) | 4.5 (1.4) | 1.5* (1.4-1.7) | <.001 | 1.0 (0.9-1.2) | 0.764 |
|  | *Attempt* | 1.4 (0.0) | 3.7 (0.4) | 4.5 (0.7) | 7.2 (1.3) | 5.8 (1.6) | 1.6* (1.5-1.7) | <.001 | 1.2* (1.0-1.4) | 0.029 |

**Supplemental table 10.** Lifetime prevalence of suicidality according to number of specific phobia subtypes, among participants *with* current psychopathology

| **Category** | **Subcategory** | **Number of subtypes** | | | | | **Test of linear effect**  **(total sample)** | | **Test of linear effect**  **(SP cases only)** | |
| --- | --- | --- | --- | --- | --- | --- | --- | --- | --- | --- |
|  |  | **0** | **1** | **2** | **3** | **4+** |  |  |  |  |
|  | | *% (SE)* | *% (SE)* | *% (SE)* | *% (SE)* | *% (SE)* | *OR (95% CI)* | *p-value* | *OR (95% CI)* | *p-value* |
| **Suicidality** | *Ideation* | 28.3 (0.6) | 36.1 (1.7) | 37.1 (2.0) | 42.2 (2.8) | 43.7 (3.4) | 1.2* (1.1-1.2) | <.001 | 1.1* (1.0-1.2) | 0.016 |
|  | *Plan* | 11.8 (0.4) | 13.3 (1.2) | 16.8 (1.5) | 21.7 (2.4) | 20.8 (2.7) | 1.2* (1.1-1.3) | <.001 | 1.2* (1.1-1.4) | <.001 |
|  | *Attempt* | 10.4 (0.4) | 14.1 (1.1) | 14.3 (1.5) | 19.8 (2.2) | 24.5 (2.7) | 1.2* (1.2-1.3) | <.001 | 1.2* (1.1-1.4) | <.001 |

**Supplemental table 11.** Prevalence and characteristics of specific phobia, among participants from low or middle income countries

|  | **Any specific phobia** | **Any early-onset specific phobia** | **Number of subtypes** | | | | **Test of linear effect** | |
| --- | --- | --- | --- | --- | --- | --- | --- | --- |
|  |  |  | **1** | **2** | **3** | **4+** |  | |
|  | *% (SE)* | *% (SE)* | *% (SE)* | *% (SE)* | *% (SE)* | *% (SE)* | *OR (95% CI)* | *P-value* |
| **Lifetime prevalence** | 6.5 (0.1) | 5.1 (0.1) | 2.8 (0.1) | 1.3 (0.1) | 0.5 (0.0) | 0.4 (0.0) | - | - |
| **12-month prevalence** | 4.9 (0.1) | 3.9 (0.1) | 2.1 (0.1) | 1.0 (0.1) | 0.4 (0.0) | 0.4 (0.0) | - | - |
| **Persistence** | 76.0 (0.9) | 76.4 (1.0) | 74.7 (1.4) | 77.3 (2.0) | 77.1 (3.0) | 83.8 (2.9) | 1.2* (1.0-1.4) | 0.008 |
| **Severe disability** | 18.0 (0.9) | 18.5 (0.9) | 16.3 (1.3) | 21.0 (1.9) | 24.2 (3.4) | 18.2 (3.0) | 1.1 (1.0-1.3) | 0.087 |
| **Treatment for specific phobia** | 13.8 (0.7) | 12.7 (0.8) | 11.2 (0.9) | 15.6 (1.8) | 12.7 (1.9) | 14.2 (2.6) | 1.1 (1.0-1.3) | 0.175 |

**Supplemental table 12.** Prevalence and characteristics of specific phobia, among participants from high income countries

|  | **Any specific phobia** | **Any early-onset specific phobia** | **Number of subtypes** | | | | **Test of linear effect** | |
| --- | --- | --- | --- | --- | --- | --- | --- | --- |
|  |  |  | **1** | **2** | **3** | **4+** |  | |
|  | *% (SE)* | *% (SE)* | *% (SE)* | *% (SE)* | *% (SE)* | *% (SE)* | *OR (95% CI)* | *P-value* |
| **Lifetime prevalence** | 8.7 (0.2) | 6.6 (0.1) | 3.7 (0.1) | 1.7 (0.1) | 0.7 (0.0) | 0.5 (0.0) | - | - |
| **12-month prevalence** | 6.4 (0.1) | 4.9 (0.1) | 2.7 (0.1) | 1.3 (0.1) | 0.6 (0.0) | 0.4 (0.0) | - | - |
| **Persistence** | 73.1 (0.8) | 74.3 (0.9) | 72.4 (1.2) | 73.6 (1.7) | 77.9 (2.2) | 86.4 (2.1) | 1.3* (1.1-1.4) | <.001 |
| **Severe disability** | 19.8 (0.8) | 20.6 (0.9) | 18.3 (1.1) | 19.6 (1.9) | 23.9 (2.4) | 33.6 (3.4) | 1.4* (1.2-1.5) | <.001 |
| **Treatment for specific phobia** | 28.7 (0.7) | 26.8 (0.8) | 25.1 (1.1) | 28.5 (1.5) | 29.1 (2.6) | 30.7 (2.9) | 1.2* (1.0-1.3) | 0.011 |

**Supplemental table 13.** Prevalence of comorbid internalizing disorders, as a function of number of specific phobia subtypes, among participants from low or middle income countries

| **Comorbid disorder** | **Number of subtypes** | | | | | **Test of linear effect**  **(total sample)** | | **Test of linear effect**  **(SP cases only)** | |
| --- | --- | --- | --- | --- | --- | --- | --- | --- | --- |
|  | **0** | **1** | **2** | **3** | **4+** |  |  |  |  |
|  | *% (SE)* | *% (SE)* | *% (SE)* | *% (SE)* | *% (SE)* | *OR (95% CI)* | *p-value* | *OR (95% CI)* | *p-value* |
| Agoraphobia | 0.6 (0.1) | 4.7 (0.7) | 9.1 (1.3) | 18.6 (3.4) | 20.2 (3.8) | 2.7* (2.4-3.0) | <.001 | 1.8* (1.5-2.1) | <.001 |
| Generalized anxiety disorder | 2.0 (0.1) | 5.0 (0.6) | 8.3 (1.3) | 14.2 (2.6) | 16.9 (4.2) | 1.9* (1.7-2.1) | <.001 | 1.6* (1.3-2.0) | <.001 |
| Panic disorder | 0.8 (0.1) | 3.2 (0.6) | 4.4 (0.8) | 4.8 (1.4) | 8.2 (1.9) | 1.9* (1.7-2.1) | <.001 | 1.4* (1.1-1.7) | 0.004 |
| Post-traumatic stress disorder | 1.7 (0.1) | 4.5 (0.5) | 6.0 (1.2) | 8.7 (2.2) | 11.7 (2.7) | 1.7* (1.6-1.9) | <.001 | 1.4* (1.1-1.6) | <.001 |
| Separation anxiety disorder | 3.8 (0.2) | 12.6 (1.3) | 19.8 (2.4) | 26.7 (3.9) | 16.6 (2.8) | 1.8* (1.7-1.9) | <.001 | 1.3* (1.1-1.5) | 0.002 |
| Social phobia | 1.7 (0.1) | 9.6 (0.9) | 18.1 (1.9) | 23.4 (3.6) | 29.2 (4.2) | 2.4* (2.2-2.6) | <.001 | 1.5* (1.3-1.8) | <.001 |
| **Any anxiety disorder** | 7.2 (0.2) | 25.3 (1.5) | 38.7 (2.7) | 55.7 (4.1) | 53.6 (4.7) | 2.3* (2.2-2.5) | <.001 | 1.6* (1.4-1.9) | <.001 |
| Major depression/dysthymia | 7.0 (0.2) | 18.4 (1.4) | 24.0 (2.1) | 30.6 (4.0) | 30.1 (4.5) | 1.7* (1.6-1.8) | <.001 | 1.3* (1.1-1.4) | <.001 |
| Bipolar disorder | 1.1 (0.1) | 3.2 (0.5) | 10.4 (1.6) | 9.2 (2.0) | 11.4 (2.4) | 2.0* (1.9-2.2) | <.001 | 1.5* (1.3-1.8) | <.001 |
| **Any mood disorder** | 8.0 (0.2) | 21.6 (1.4) | 34.4 (2.3) | 39.8 (4.2) | 41.5 (4.9) | 1.9* (1.8-2.0) | <.001 | 1.4* (1.2-1.6) | <.001 |
| Bulimia nervosa | 0.5 (0.1) | 1.4 (0.4) | 3.7 (1.2) | 6.0 (3.0) | 4.5 (1.9) | 2.0* (1.6-2.4) | <.001 | 1.9* (1.4-2.5) | <.001 |
| Binge eating disorder | 1.3 (0.1) | 5.6 (1.4) | 4.8 (1.3) | 7.3 (3.1) | 4.3 (2.0) | 1.6* (1.4-1.9) | <.001 | 1.1 (0.8-1.4) | 0.552 |
| **Any eating disorder** | 1.7 (0.1) | 6.9 (1.3) | 8.3 (1.6) | 12.9 (3.7) | 8.6 (2.7) | 1.8* (1.6-2.1) | <.001 | 1.4* (1.1-1.7) | 0.002 |
| **Any internalizing disorder** | 13.0 (0.3) | 38.1 (1.8) | 53.9 (2.7) | 70.7 (3.7) | 65.9 (4.6) | 2.4* (2.2-2.6) | <.001 | 1.6* (1.4-1.9) | <.001 |
| Exactly 1 INT disorder | 9.6 (0.2) | 23.6 (1.6) | 28.5 (2.4) | 31.5 (3.8) | 24.7 (4.0) | 1.5* (1.4-1.6) | <.001 | 1.1 (1.0-1.2) | 0.228 |
| Exactly 2 INT disorders | 2.3 (0.1) | 9.1 (1.0) | 13.2 (1.8) | 21.5 (3.6) | 17.9 (3.0) | 1.9* (1.8-2.1) | <.001 | 1.3* (1.1-1.6) | 0.001 |
| Exactly 3 INT disorders | 0.8 (0.1) | 3.6 (0.6) | 6.9 (1.2) | 10.3 (2.4) | 12.1 (2.3) | 2.1* (1.9-2.3) | <.001 | 1.5* (1.3-1.8) | <.001 |
| 4+ INT disorders | 0.3 (0.0) | 1.8 (0.4) | 5.4 (1.0) | 7.5 (2.1) | 11.2 (3.5) | 2.7* (2.4-3.2) | <.001 | 1.9* (1.5-2.6) | <.001 |

**Supplemental table 14.** Prevalence of comorbid internalizing disorders, as a function of number of specific phobia subtypes, among participants from high income countries

| **Comorbid disorder** | **Number of subtypes** | | | | | **Test of linear effect**  **(total sample)** | | **Test of linear effect**  **(SP cases only)** | |
| --- | --- | --- | --- | --- | --- | --- | --- | --- | --- |
|  | **0** | **1** | **2** | **3** | **4+** |  |  |  |  |
|  | *% (SE)* | *% (SE)* | *% (SE)* | *% (SE)* | *% (SE)* | *OR (95% CI)* | *p-value* | *OR (95% CI)* | *p-value* |
| Agoraphobia | 0.9 (0.1) | 5.4 (0.5) | 9.8 (1.1) | 16.6 (2.4) | 22.1 (2.8) | 2.5* (2.4-2.7) | <.001 | 1.7* (1.5-1.9) | <.001 |
| Generalized anxiety disorder | 4.9 (0.1) | 13.1 (0.8) | 18.4 (1.5) | 25.5 (2.4) | 25.2 (3.2) | 1.8* (1.7-1.9) | <.001 | 1.3* (1.2-1.4) | <.001 |
| Panic disorder | 2.0 (0.1) | 7.2 (0.7) | 11.9 (1.1) | 17.7 (2.3) | 22.4 (2.8) | 2.1* (2.0-2.2) | <.001 | 1.5* (1.3-1.7) | <.001 |
| Post-traumatic stress disorder | 3.8 (0.1) | 11.8 (0.9) | 21.2 (1.7) | 24.1 (2.4) | 30.5 (3.3) | 2.0* (1.9-2.1) | <.001 | 1.4* (1.3-1.6) | <.001 |
| Separation anxiety disorder | 4.0 (0.2) | 12.9 (1.2) | 19.7 (2.4) | 27.5 (3.1) | 38.7 (4.2) | 2.0* (1.8-2.1) | <.001 | 1.5* (1.3-1.6) | <.001 |
| Social phobia | 4.8 (0.1) | 18.7 (1.2) | 26.8 (1.7) | 36.5 (2.9) | 46.1 (3.7) | 2.2* (2.1-2.3) | <.001 | 1.5* (1.3-1.6) | <.001 |
| **Any anxiety disorder** | 13.8 (0.3) | 39.6 (1.5) | 52.9 (2.3) | 66.9 (2.7) | 77.0 (3.1) | 2.4* (2.3-2.6) | <.001 | 1.6* (1.5-1.8) | <.001 |
| Major depression/dysthymia | 13.9 (0.2) | 28.2 (1.2) | 34.5 (1.9) | 39.8 (3.2) | 37.6 (3.1) | 1.6* (1.5-1.7) | <.001 | 1.2* (1.1-1.3) | <.001 |
| Bipolar disorder | 2.8 (0.1) | 8.2 (0.9) | 9.7 (1.1) | 15.3 (2.2) | 22.7 (2.9) | 1.8* (1.7-1.9) | <.001 | 1.4* (1.3-1.6) | <.001 |
| **Any mood disorder** | 15.9 (0.3) | 34.0 (1.4) | 42.7 (2.0) | 53.0 (3.0) | 59.0 (3.5) | 1.8* (1.7-1.9) | <.001 | 1.4* (1.3-1.5) | <.001 |
| Bulimia nervosa | 0.7 (0.1) | 2.3 (0.5) | 3.5 (0.8) | 7.3 (1.7) | 6.9 (2.3) | 2.0* (1.7-2.2) | <.001 | 1.6* (1.2-2.0) | <.001 |
| Binge eating disorder | 1.4 (0.1) | 5.7 (1.1) | 5.5 (1.1) | 6.1 (2.0) | 7.7 (2.5) | 1.7* (1.5-1.8) | <.001 | 1.0 (0.8-1.3) | 0.946 |
| **Any eating disorder** | 2.0 (0.1) | 7.4 (1.1) | 8.3 (1.3) | 12.0 (2.3) | 13.3 (3.2) | 1.8* (1.7-1.9) | <.001 | 1.2 (1.0-1.5) | 0.072 |
| **Any internalizing disorder** | 23.4 (0.4) | 52.2 (1.7) | 66.2 (2.3) | 78.7 (2.6) | 84.0 (2.7) | 2.4* (2.3-2.6) | <.001 | 1.7* (1.5-1.9) | <.001 |
| Exactly 1 INT disorder | 15.0 (0.3) | 24.3 (1.2) | 27.0 (1.8) | 23.7 (2.6) | 18.9 (2.7) | 1.2* (1.2-1.3) | <.001 | 1.0 (0.9-1.1) | 0.582 |
| Exactly 2 INT disorders | 5.6 (0.2) | 14.4 (0.9) | 16.6 (1.5) | 21.7 (2.2) | 21.2 (3.1) | 1.6* (1.5-1.7) | <.001 | 1.2* (1.0-1.3) | 0.006 |
| Exactly 3 INT disorders | 1.9 (0.1) | 7.6 (0.6) | 10.9 (1.2) | 14.5 (1.8) | 17.3 (2.7) | 1.9* (1.8-2.0) | <.001 | 1.3* (1.2-1.5) | <.001 |
| 4+ INT disorders | 0.9 (0.1) | 5.9 (0.6) | 11.6 (1.2) | 18.9 (2.4) | 26.5 (3.6) | 2.6* (2.4-2.7) | <.001 | 1.7* (1.5-1.9) | <.001 |

**Supplemental table 15.** Lifetime prevalence of suicidality according to number of specific phobia subtypes, among participants from low or middle income countries

| **Category** | **Subcategory** | **Number of subtypes** | | | | | **Test of linear effect**  **(total sample)** | | **Test of linear effect**  **(SP cases only)** | |
| --- | --- | --- | --- | --- | --- | --- | --- | --- | --- | --- |
|  |  | **0** | **1** | **2** | **3** | **4+** |  |  |  |  |
|  | | *% (SE)* | *% (SE)* | *% (SE)* | *% (SE)* | *% (SE)* | *OR (95% CI)* | *p-value* | *OR (95% CI)* | *p-value* |
| **Suicidality** | *Ideation* | 5.3 (0.1) | 14.6 (1.1) | 17.8 (2.0) | 25.4 (2.8) | 25.8 (3.4) | 1.6* (1.5-1.7) | <.001 | 1.2* (1.1-1.4) | <.001 |
|  | *Plan* | 1.6 (0.1) | 4.8 (0.7) | 8.1 (1.5) | 9.8 (1.7) | 11.7 (2.5) | 1.7* (1.5-1.8) | <.001 | 1.3* (1.1-1.6) | 0.002 |
|  | *Attempt* | 1.5 (0.1) | 4.9 (0.6) | 6.6 (1.1) | 10.5 (1.8) | 13.1 (2.3) | 1.7* (1.6-1.8) | <.001 | 1.4* (1.2-1.6) | <.001 |
| **Serious mental illness** |  | 2.6 (0.1) | 10.2 (1.0) | 20.2 (1.9) | 27.2 (3.7) | 26.1 (4.1) | 2.2* (2.0-2.4) | <.001 | 1.6* (1.3-1.8) | <.001 |

**Supplemental table 16.** Lifetime prevalence of suicidality according to number of specific phobia subtypes, among participants from high income countries

| **Category** | **Subcategory** | **Number of subtypes** | | | | | **Test of linear effect**  **(total sample)** | | **Test of linear effect**  **(SP cases only)** | |
| --- | --- | --- | --- | --- | --- | --- | --- | --- | --- | --- |
|  |  | **0** | **1** | **2** | **3** | **4+** |  |  |  |  |
|  | | *% (SE)* | *% (SE)* | *% (SE)* | *% (SE)* | *% (SE)* | *OR (95% CI)* | *p-value* | *OR (95% CI)* | *p-value* |
| **Suicidality** | *Ideation* | 9.6 (0.2) | 22.0 (1.0) | 28.1 (1.6) | 34.4 (2.6) | 37.2 (3.2) | 1.7* (1.6-1.7) | <.001 | 1.3* (1.2-1.4) | <.001 |
|  | *Plan* | 2.9 (0.1) | 8.2 (0.6) | 10.6 (1.0) | 16.2 (2.0) | 16.2 (2.4) | 1.7* (1.6-1.8) | <.001 | 1.3* (1.2-1.5) | <.001 |
|  | *Attempt* | 2.6 (0.1) | 7.7 (0.6) | 9.8 (1.0) | 15.4 (1.8) | 20.1 (2.5) | 1.8* (1.7-1.9) | <.001 | 1.4* (1.2-1.5) | <.001 |
| **Serious mental illness** |  | 4.4 (0.1) | 14.5 (1.0) | 21.4 (1.6) | 28.1 (2.5) | 46.0 (3.4) | 2.1* (2.0-2.2) | <.001 | 1.5* (1.4-1.7) | <.001 |
